# Exploring the Boundaries of Aromaticity in Complex Structures and Excited States Through Computational Analysis

**DOI:** 10.1002/chem.71596

**Published:** 2026-08-21

**Authors:** Sílvia Escayola, Miquel Solà, Albert Poater

**Affiliations:** ^1^ Departament de Química Inorgànica i Orgànica Universitat de Barcelona Barcelona Catalonia Spain; ^2^ Institut De Química Computacional i Catàlisi and Departament de Química Universitat de Girona Girona Catalonia Spain

**Keywords:** aromaticity, DFT, electron delocalization, excited state, functional materials

## Abstract

Aromaticity is a central concept for understanding and predicting molecular structure, reactivity, and properties. Commonly associated with cyclic or 3D systems, it originates from electron delocalization along a closed pathway, producing characteristic effects such as enhanced thermodynamic stability, magnetic anisotropy, and unusual chemical shifts. Although aromaticity is not a directly measurable physical observable, it has remained a key concept in chemistry for almost two centuries. Its counterpart, antiaromaticity, is generally linked to reduced stability and increased reactivity, further emphasizing the importance of electron delocalization in molecular behavior. In recent years, aromaticity has gained particular relevance in the study of electronically excited states, where it helps explain photochemical behavior and guides the development of advanced materials and reactions. The extension of ground‐state aromaticity principles to excited‐state chemistry has proven promising, but uncertainties remain regarding the applicability of specific aromaticity rules and descriptors in complex excited systems. At the same time, the concept of aromaticity has expanded far beyond classical annulenes. Modern examples include molecules with multiple delocalization pathways, aromatic character involving σ‐, π‐, δ‐, or ϕ‐electrons, and nonplanar or 3D architectures, challenging conventional definitions and motivating renewed conceptual reassessment. These advances continue reshaping theoretical perspectives on molecular delocalization phenomena worldwide.

## Graphical abstract

Understanding aromaticity has evolved from benzene‐centric concepts to a broad multidimensional framework spanning organic, inorganic, and excited‐state systems. This review highlights ambiguities between magnetic, electronic, geometric, and energetic descriptors, emphasizing the need for unified, multi‐criteria approaches. Future advances combining computation, spectroscopy, and machine learning will enable predictive design of functional materials.

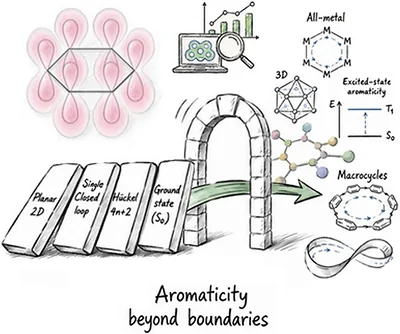

## 1 Introduction

Humans possess an inherent desire to comprehend the world around them. Whether rooted in survival instincts, cultural development, or intellectual curiosity, this drive is accompanied by a remarkable imaginative capacity that distinguishes our species. Since the late Pliocene, approximately 2.5 million years ago, human beings have progressively developed the ability to investigate their surroundings, communicate ideas, and construct increasingly abstract interpretations of reality. Over time, Homo sapiens acquired exceptional communicative skills, creativity, and the ability to recognize both intuitive and conceptual relationships. These capabilities expanded dramatically with the transition from prehistory to recorded history, when written language enabled the preservation and transmission of accumulated knowledge and experiences. This turning point initiated a continuous process of intellectual and technological advancement that persists today.

“Science is organized knowledge.”

— attributed to Immanuel Kant

As information became increasingly organized and transferable among generations, the conceptual foundations of modern natural sciences gradually emerged. Early civilizations in ancient Egypt and Mesopotamia carried out systematic observations of natural and physical phenomena, while philosophers from Greek, Indian, and other intellectual traditions sought to answer a profound question: what constitutes reality itself? Among the proposed explanations was the idea that matter consists of indivisible microscopic units. In ancient Greece, Leucippus and Democritus introduced the term atomos, meaning “indivisible” or “uncuttable,” to describe these fundamental particles [1]. Nevertheless, a rigorous scientific atomic theory did not gain widespread acceptance until the early nineteenth century, when John Dalton proposed, based on experimental evidence, that chemical substances decompose into combinations of elements in fixed and reproducible proportions [2].

Following the establishment of atomic theory, the identification of an ever‐expanding number of chemical elements created the need for a structured system capable of organizing this emerging knowledge. This necessity ultimately gave rise to the periodic table of elements (Figure 1), arguably the most recognizable emblem of chemistry and one of the discipline's greatest achievements in scientific classification. In its modern form, the periodic table developed by Henry Moseley organizes elements according to atomic number, electronic structure, and recurring physicochemical properties. Building upon earlier classification efforts and major contributions from figures such as Antoine Lavoisier, Johann Wolfgang Döbereiner, and Dmitri Mendeleev, this framework established a coherent system for understanding elemental relationships [3]. Beyond enabling the systematic classification of known elements during the nineteenth century, it also demonstrated remarkable predictive power by anticipating the existence and properties of elements that had not yet been experimentally identified.

**FIGURE 1:**
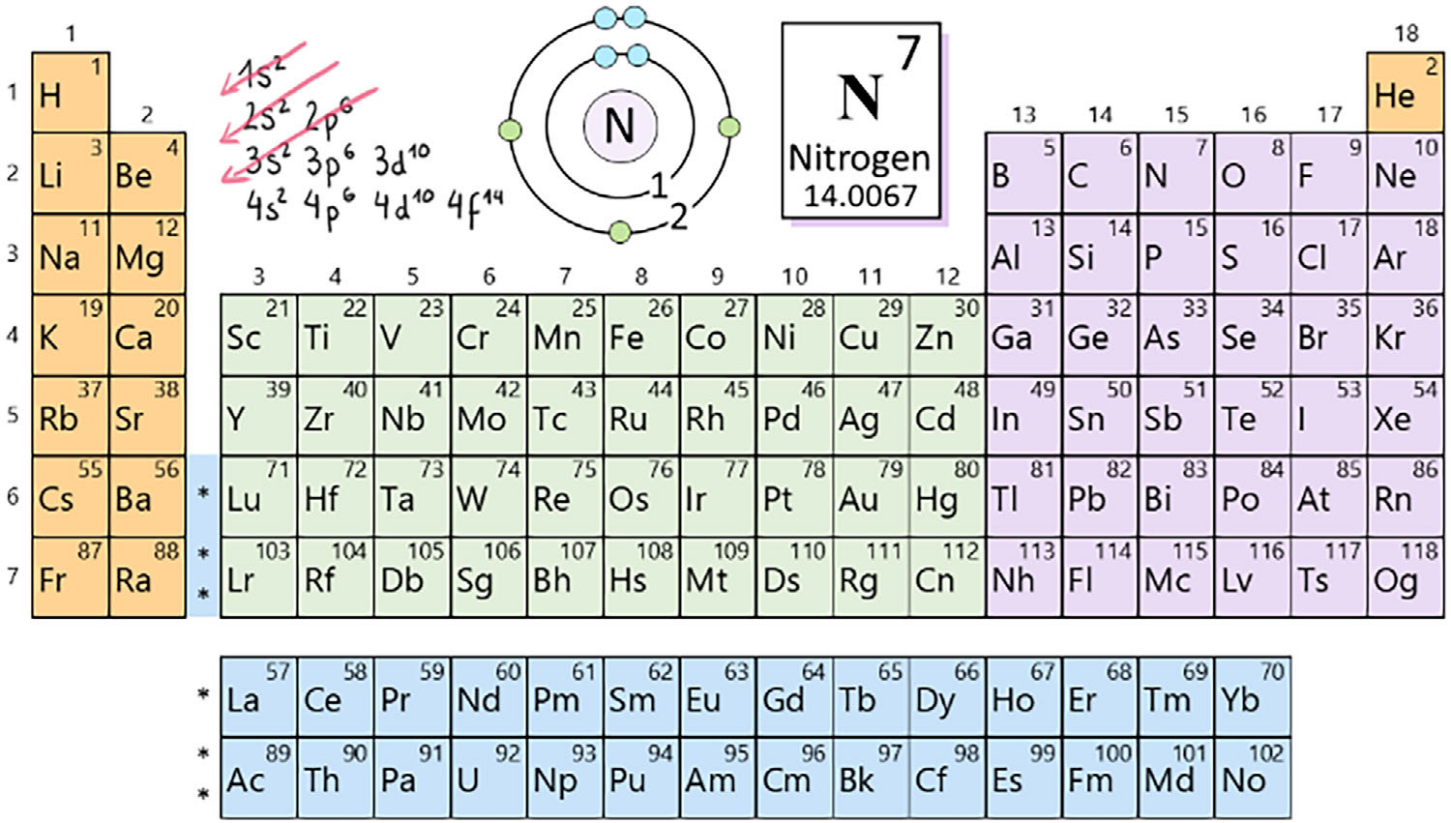
Periodic table of the chemical elements, accompanied at the top by a simplified Moeller diagram and a Bohr–Rutherford representation of the nitrogen atom.

Although the periodic table constitutes the principal framework for organizing chemical elements, symmetry represents an equally essential principle for the classification and interpretation of molecular structures, properties, and reactivity. Notably, symmetry is far from being exclusive to chemistry, as it emerges across diverse disciplines, natural phenomena, and theoretical frameworks. A striking illustration of this interdisciplinary role is the discovery of Buckminsterfullerene (C_60_), depicted in Figure 2a, where symmetry established a conceptual bridge between chemistry and architecture.

**FIGURE 2:**
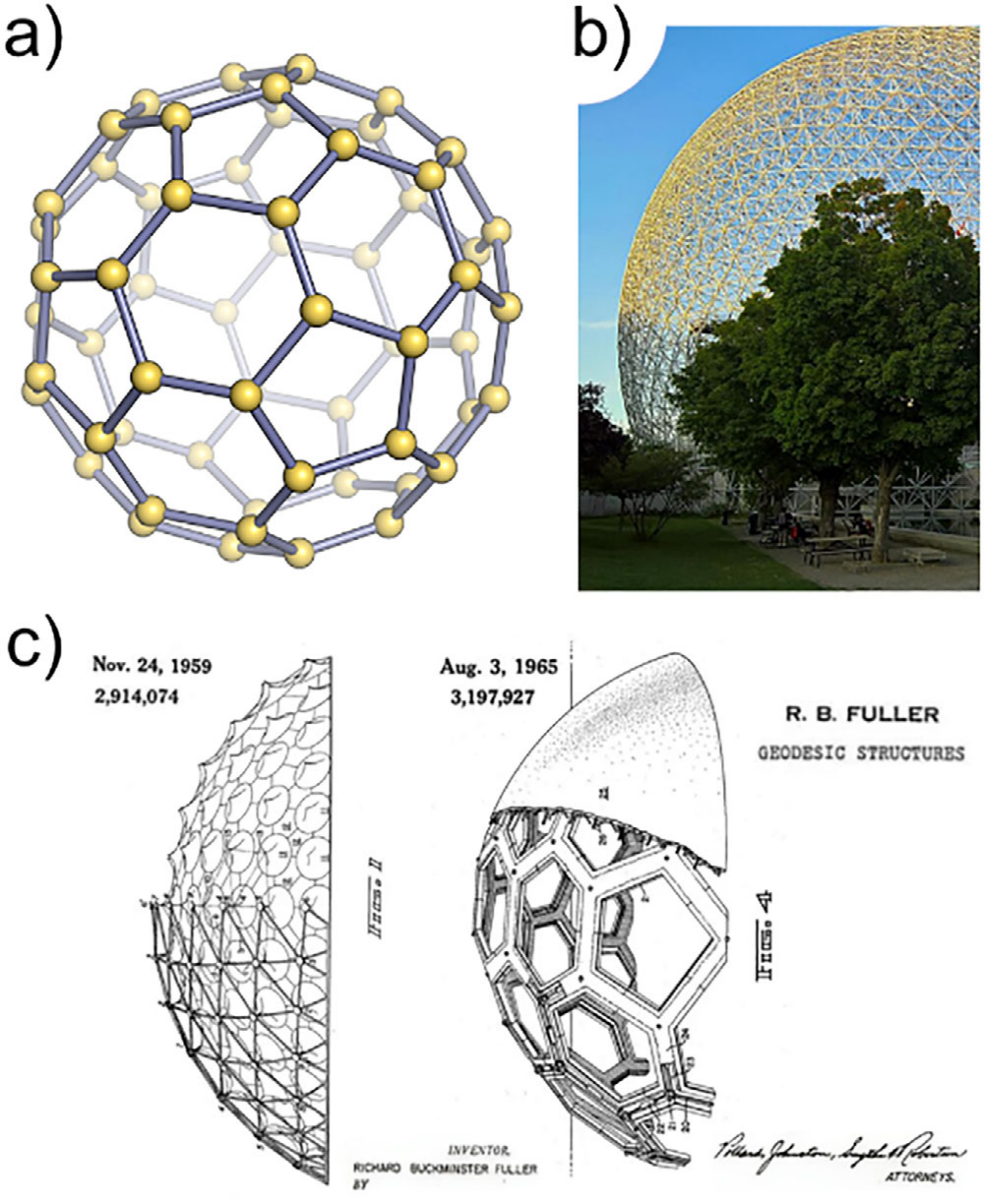
Structure of fullerenes and geodesic domes. (a) Molecular structure of C_60_. (b) The Montreal Biosphère Museum, a representative example of geodesic dome architecture originally built for the 1967 International and Universal Exposition. Photograph by Andre Carrotflower, adapted by cropping from the original available via Wikimedia Commons, licensed under CC BY‐SA 4.0 [7]. (c) Original design drawings of Buckminster Fuller's geodesic domes, adapted from Ref. [8].

In 1985, Harold Kroto, Robert Curl, Richard Smalley, and collaborators reported the landmark discovery that would ultimately earn them the Nobel Prize in Chemistry eleven years later [4]. Although a molecular species corresponding to C_60_ had previously been observed by mass spectrometry [5], its precise structural identity remained unresolved. The breakthrough came when the characteristic mass signal at m/z 720 was interpreted as an icosahedral carbon framework, inspired by the geometric principles underlying the architectural works of Buckminster Fuller. Fuller, while not the original inventor, popularized the concept of the geodesic dome and designed several emblematic examples, including the Montreal Biosphère (Figure 2b,c), whose geometry closely resembles the highly symmetric structure of C_60_ [6].

This example illustrates the broad and profound role of symmetry in chemistry. Beyond its usefulness in predicting molecular geometries and interpreting Lewis structures, its most powerful application lies in the use of group theory within quantum mechanics. The deep connection between group theory and quantum mechanics was established in the late 1920s through the work of Weyl, Wigner, Heisenberg, and others [9]. This framework later became especially valuable with the development of computational quantum chemistry in the second half of the 20th century, where it evolved into an essential tool for practical calculations.

The explicit use of molecular symmetry, when present, not only reduces the computational cost associated with solving equations but also provides valuable insight into molecular properties, from which structural features can be inferred. To this end, molecules are classified according to their symmetry elements and operations, namely the identity operation, n‐fold rotation axis, mirror plane, improper rotation axis, and inversion center, denoted as *E*, *C*_n_, *σ*, *S*_n_, and *i*, respectively. On this basis, the point group of a molecule can be determined from its symmetry elements. A key advantage of this classification is that, once the point group is assigned, the specific chemical complexity of the molecule becomes secondary; instead, the focus shifts to an abstract geometric object embodying the same symmetry characteristics [10]. In practice, point‐group assignment is typically carried out using a decision flow chart, such as that illustrated in Figure 3, which in the case of C_60_ leads to the *I*_h_ point group.

**FIGURE 3:**
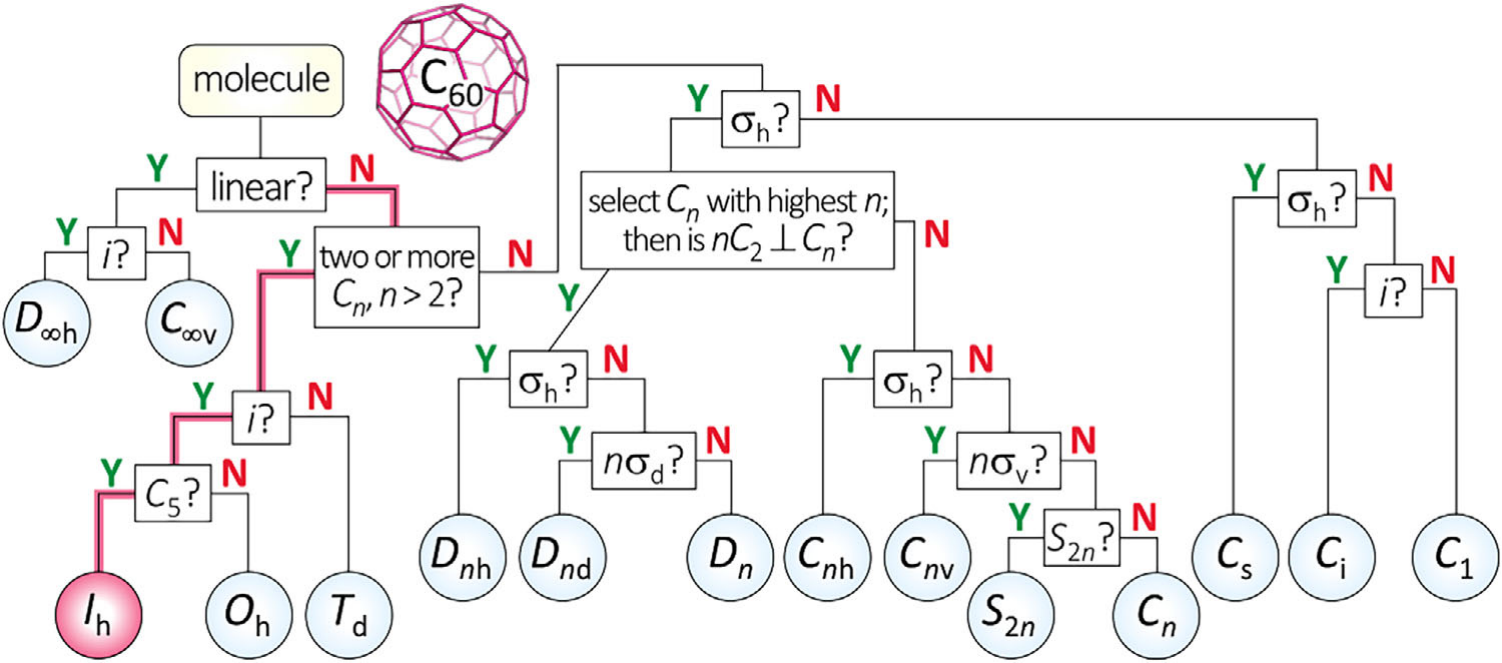
Symmetry‐based flow chart for determining the point group of a molecule, illustrated here for the case of C_60_.

The preceding examples demonstrate that classification is not merely a strategy for imposing order on seemingly disordered data; rather, it is a fundamental tool that actively guides scientific inquiry and enables meaningful progress.

In the broader context of scientific organization, and particularly in computational chemistry, it is noteworthy that the Catalan, Basque, Spanish, and French terms for “computer” derive from the Latin *ordinator*, meaning “one who orders” [11]. This etymology highlights the central role of computation in structuring and systematizing information. Furthermore, with the advent of artificial intelligence, this capacity for organization has evolved into a dynamic feedback mechanism that also enhances algorithmic learning [12]. Modern computational tools not only sort data but are increasingly capable of identifying patterns and predicting the behavior of chemical systems [13, 14, 15, 16]. In doing so, they extend the reach of traditional classification schemes and reveal subtle features that may remain inaccessible to human intuition alone [17].

This advanced level of organization and analysis is particularly important in chemistry. The comparative study of atoms, molecular fragments, or entire molecules is essential for quantifying their distinctive characteristics. Such comparisons are typically performed using either simplified models or more sophisticated computational approaches grounded in classical or quantum mechanics. Whether these quantities correspond to directly measurable observables (e.g., geometries, energies, or dipole moments) or to derived concepts that cannot be measured directly (e.g., covalent radii, bond orders, atomic charges, oxidation states or aromaticity), they remain central to understanding the nature of materials [18]. Nonobservable quantities, however, lack unique reference values and can therefore be described through multiple metrics that are not universally transferable. Despite this inherent ambiguity, their practical usefulness, particularly in facilitating communication within the chemical community, has been widely demonstrated [19, 20, 21, 22], motivating ongoing efforts to refine their definitions and extend their applicability [23].

As noted above, computational chemistry relies on computer‐based simulations [24], representing a paradigm shift in the analysis and interpretation of chemical data. It moves beyond traditional approaches by transforming molecular classification and analysis into predictive and mechanistically insightful tools [25, 26, 27, 28]. This discipline spans a broad range of topics and influences virtually all areas of chemistry, providing detailed understanding of both experimental observations and theoretical frameworks. In the following, the focus of the review is narrowed to one specific area: aromaticity as a descriptor for characterizing complex molecular systems in their ground and low‐lying excited states. We examine whether aromatic character correlates with specific molecular properties, while also assessing the strengths and limitations of different aromaticity indicators [29]. In addition, we provide design guidelines aimed at achieving targeted aromatic features in molecular systems. Through this work, we aim to contribute to a more robust classification of chemical systems, thereby supporting further advances in practical applications within the field.

## 2 The Background of Aromaticity

### 2.1 The Concept of Aromaticity

Aromaticity is a long‐standing concept in chemistry that is central to understanding both the electronic structure and the reactivity of molecules. It is typically associated with cyclic systems that exhibit enhanced stability due to electron delocalization and that often display distinctive magnetic and electronic properties. Although aromaticity was originally introduced to rationalize benzene‐like compounds and their unusual stability, the concept, together with its counterpart antiaromaticity, has since been extended to a wide range of systems, including heterocycles, inorganic rings exhibiting σ‐, π‐, δ‐, or ϕ‐aromaticity (or combinations thereof), as well as to molecules with Möbius topology [30] and 3D molecular architectures [31]. Moreover, aromaticity is no longer restricted to ground states; it also encompasses excited states [32] and has been invoked in transition states [33], extending its relevance beyond stable compounds and introducing additional conceptual and methodological challenges that remain only partially resolved. Given these complexities, arising from both molecular topology and the specific electronic state considered, and the large diversity of available descriptors and evaluation criteria, a careful and critical analysis is required to properly rationalize experimental observations [34]. In this section, we review the historical development of the concept, the distinction between intrinsic and response aromaticity, the current state of the field, and the main challenges, particularly in excited‐state systems and topologically complex molecules.

### 2.2 The History of the Concept of Aromaticity

The history of aromaticity begins with the discovery and early characterization of benzene. In 1825, Michael Faraday (Figure 4) isolated a compound from an oily residue used in lamps, which exhibited a simple 1:1 carbon‐to‐hydrogen ratio and which he termed “bicarburet of hydrogen” [35, 36]. A few years later, Eilhard Mitscherlich synthesized the same compound by heating benzoic acid derived from benzoin, naming it “benzin” [37], and establishing its empirical formula as C_6_H_6_. During the nineteenth century, numerous related compounds were discovered, many of which shared low C/H ratios and characteristic odors (Figure 5), leading August Wilhelm von Hofmann to introduce the term “aromatic” to describe this class of substances [38].

**FIGURE 4:**
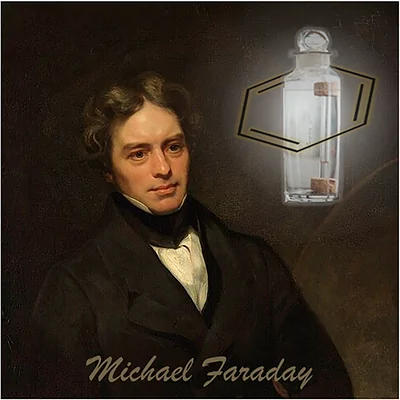
Portrait of Michael Faraday by Thomas Phillips (1841–1842), National Portrait Gallery, London, NPG 269. Public‐domain image from Wikimedia Commons, adapted by the authors. Images adapted from Ref. [39].

**FIGURE 5:**
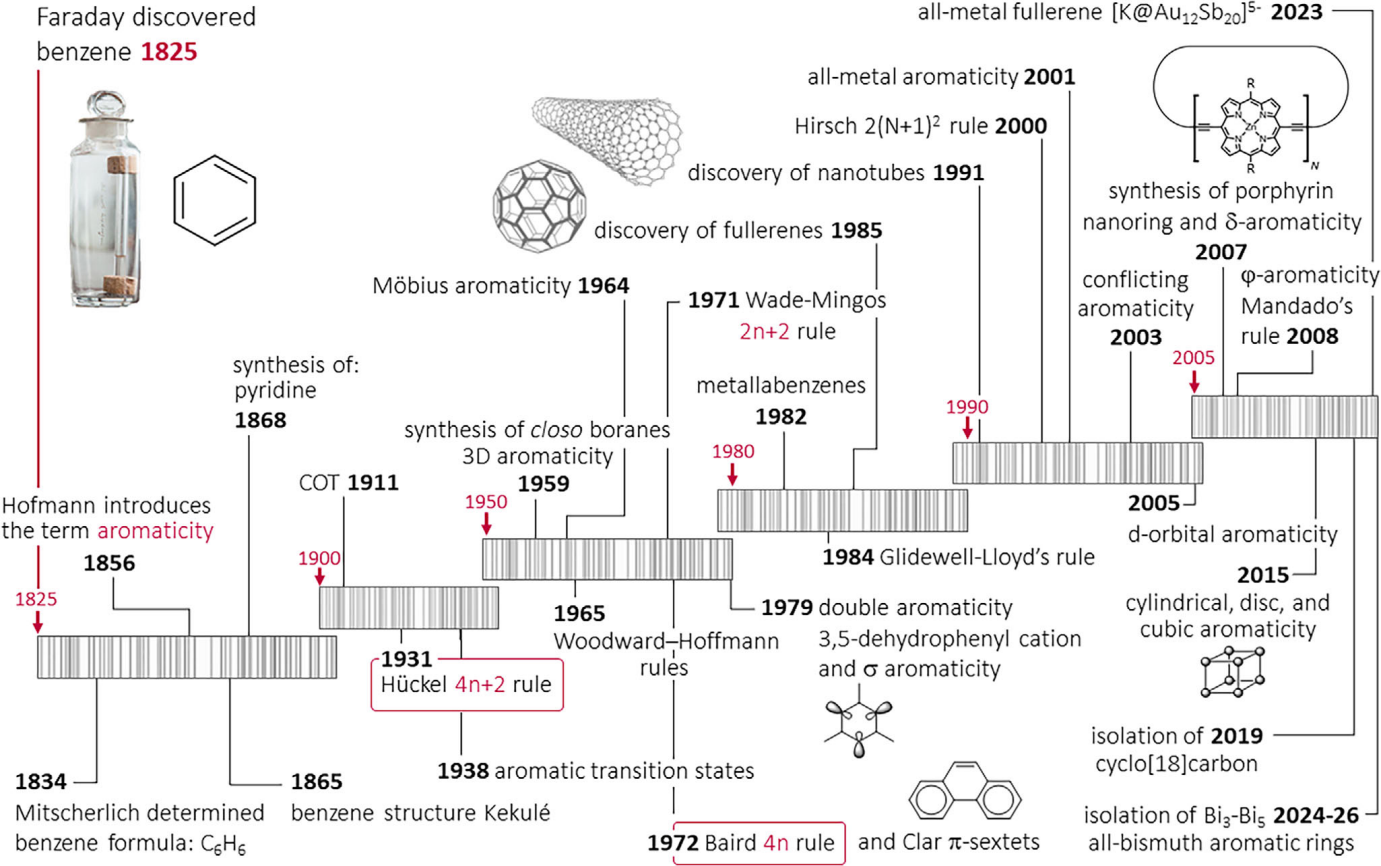
A chronological representation of aromaticity, illustrating key milestones and discoveries that have progressively shaped the concept, from its origins to the state of the art.

Despite the growing body of experimental evidence, the structure of benzene remained controversial until 1865, when Friedrich August Kekulé proposed a cyclic arrangement with alternating single and double bonds [40]. This milestone was later commemorated at the “Benzolfest,” marking 25 years since his seminal contribution. At this event, Kekulé famously recounted his dream of the Ouroboros, the serpent biting its own tail, as inspiration for the cyclic structure of benzene [41].

As the number of known aromatic compounds increased, the concept of aromaticity evolved from a benzene‐centered structural analogy to a broader classification based on chemical similarity, particularly reactivity patterns. The synthesis of heteroaromatic systems such as pyridine [42], together with polycyclic hydrocarbons like naphthalene [43], highlighted the need to refine the criteria defining aromatic behavior. Furthermore, Kekulé’s original model was insufficient to explain key experimental observations. For example, it predicted two isomers of 1,2‐disubstituted benzene, whereas only one is observed. This discrepancy led Kekulé to propose a dynamic resonance description involving two interconverting structures, introducing the idea of delocalized π‐electrons [31, 44].

Comparisons between aromatic compounds and their aliphatic analogues further supported the hypothesis that enhanced stability arises from cyclic delocalization. However, the discovery and characterization of cyclooctatetraene (COT), an [8]annulene with two resonance forms analogous to benzene, revealed that it behaves more like a noncyclic olefinic system than an aromatic one [45]. This observation demonstrated that a simple resonance picture is insufficient to fully account for aromatic stabilization.

A major conceptual breakthrough came with the development of quantum mechanics [46] and molecular orbital (MO) theory [47], which transformed the understanding of chemical bonding and molecular structure. In particular, Hückel molecular orbital (HMO) theory [48] provided a rigorous explanation for aromaticity in benzene, a fully delocalized, symmetric system without distinguishable single and double bonds, while rationalizing the nonaromatic character of systems such as COT. In 1931, Hückel put the basis of his now‐famous rule (formulated later by Doering and Detert [49]), stating that monocyclic, planar, fully conjugated systems are aromatic [50, 51, 52, 53] if they contain 4n + 2 π‐electrons and antiaromatic if they contain 4n π‐electrons (where n = 0, 1, 2, 3, …) [49, 54, 55]. In practice, many larger annulenes and flexible ring systems avoid antiaromaticity by adopting nonplanar conformations that reduce energettic destabilization.

Advances in electronic structure theory and experimental techniques progressively broadened the scope of aromaticity to include macromolecular systems [56, 57, 58, 59, 60], inorganic frameworks [61, 62, 63, 64, 65, 66, 67, 68, 69, 70, 71], 3D aromaticity [72, 73, 74, 75, 76], and systems involving σ‐, π‐, and φ‐electron delocalization [66, 77, 78, 79]. The concept was further extended to excited states, most notably through Baird's rule (1972) [80], which describes aromaticity reversal in the lowest ππ* excited states [32, 81, 82, 83, 84, 85, 86, 87].

Today, despite nearly two centuries of development, aromaticity remains a central yet formally undefined concept [34, 88, 89, 90]. Nevertheless, there is broad agreement on key features such as cyclic electron delocalization and enhanced thermodynamic stability [31, 91]. Additional commonly used indicators include bond‐length equalization, pronounced magnetic anisotropy (reflected in NMR chemical shifts), and characteristic spectroscopic signatures. These manifestations are typically quantified using electronic, energetic, geometric, magnetic, and reactivity‐based indices. However, none of these provide a unique or definitive measure of aromaticity, making its rigorous and quantitative characterization an ongoing challenge.

A schematic overview of current aromaticity classifications is presented in Figure 6 [31], encompassing structural classes, spatial and orbital arrangements, electronic states (ground, excited, and transition states), and the distinction between intrinsic and response aromaticity. A wide range of methodological approaches is employed to characterize aromaticity, including energetic, magnetic, geometric, electronic, and reactivity‐based criteria, as well as different aromaticity rules [92].

**FIGURE 6:**
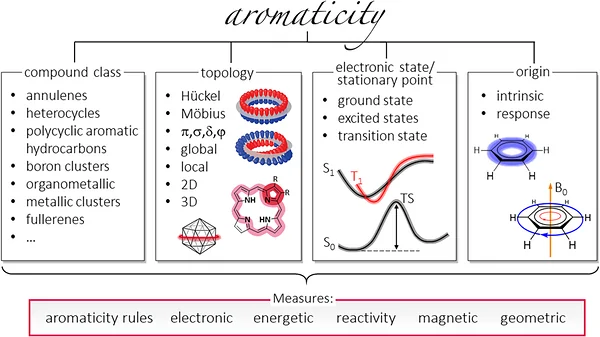
Overview of aromaticity types. The representations of Hückel and Möbius topologies are reproduced with permission from Ref. [93].

A molecule's aromatic character may fall into multiple categories simultaneously. For example, the six‐porphyrin nanoring belongs to the class of heterocyclic macrocycles and exhibits 2D π‐conjugation consistent with Hückel‐type aromaticity. Although it fulfils the formal requirements for global aromaticity, its classification remains debated. Electron delocalization indices indicate predominantly local aromaticity confined to individual porphyrin units, due to limited delocalization across the butadiyne linkers [94, 95]. In contrast, experimental NMR data and magnetic‐response calculations support global ring currents in several oxidized states. Moreover, certain systems that are not intrinsically aromatic, or are only weakly aromatic according to electronic and energetic criteria, may exhibit aromatic‐like responses under an external magnetic field (B_0_), such that their apparent aromatic character emerges or becomes more pronounced under perturbation [96, 97]. This behavior is exemplified by the most stable isomer of B_4_N_4_H_8_ (azabora‐cyclooctatetraene) in its lowest triplet state, ^3^B_4_N_4_H_8_COT‐A. Preethalayam et al. reported a global diatropic π‐ring current of 9.2 nAT^−1^ [98], approximately 60% of that of Baird‐aromatic ^3^COT, together with a remarkable nucleus‐independent chemical shifts (NICS), in particular the NICS(1)_zz_ with a value of −19.3 ppm. In contrast, its isomerization stabilization energies were close to zero and its multicenter index (MCI) was about 17 times smaller than that of ^3^COT, indicating little energetic stabilization and weak multicenter electron sharing. Together, these cases illustrate the complexity of aromaticity in modern chemical systems, and the care required when interpreting aromaticity‐related descriptors, since magnetic response aromaticity may not consistently correlate with intrinsic electron delocalization or energetic stabilization [95, 99, 100]

Such cases highlight that aromaticity, if applied uncritically, can become an ambiguous or even misleading concept. Consequently, rigorous analysis is essential. Leading experts have also noted a growing tendency to propose increasingly specialized forms of aromaticity, and have suggested that future progress may come not from continuously defining new categories, but from identifying unifying principles across aromatic systems [34, 90].

In the following sections, we discuss in more detail the distinction between intrinsic and response aromaticity, which is essential for its correct evaluation. We then address the challenges associated with excited‐state aromaticity, macrocyclic systems, and multidimensional cases such as 3D and double aromaticity, where traditional indices, originally developed for planar ground‐state molecules, may be insufficient or inapplicable.

### 2.3 Intrinsic and Response‐Based Aromaticity

The magnetic criterion of aromaticity is the most widely used approach in aromaticity studies [101]. It appears in a large fraction of the literature in this field and is often employed as the sole metric for assessing the aromatic character of a molecule. The popularity of magnetic‐based computational tools, particularly NICS [102], stems from their straightforward implementation (being readily available in most quantum chemistry packages) and their relatively intuitive interpretation, especially from an experimental perspective [103, 104, 105, 106, 107]. NICS and related magnetic descriptors aim to capture the response of delocalized σ‐, π‐, δ‐, or ϕ‐electron systems to an applied external magnetic field. Under such a field, electrons circulate around the ring, generating an induced magnetic field of their own (Figure 7a). This circulation gives rise to what is known as a ring current.

**FIGURE 7:**
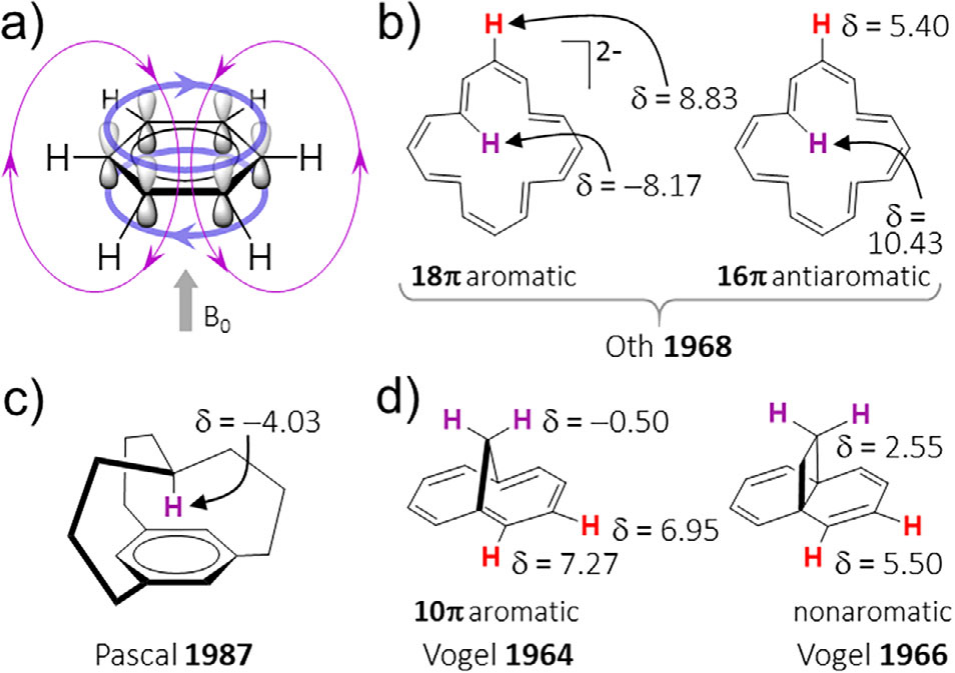
(a) Ring currents (violet), induced magnetic field (magenta), and applied magnetic field (B_0_, grey arrow). (b–d) ^1^H NMR chemical shifts (δ, ppm) for selected hydrogen atoms in (b) [16]annulene in its dianionic and neutral forms [108], (c) *in*‐[3^4,10^][7]‐metacyclophane [109], and (d) 1,6‐methano[10]annulene [110] and 1,6‐ethano[10]annulene [111]. For clarity, only the most relevant hydrogen atoms are shown.

To isolate the magnetic effects associated with this ring current, NICS values are typically evaluated at or near the geometric center of the ring [102]. This is commonly achieved by introducing a “dummy atom,” an artificial probe with no physical identity, placed at the ring centroid. The NICS value is then computed at this position [112, 113], providing a measure of magnetic shielding or deshielding induced by the circulating electron density [114, 115, 116].

Although ring currents cannot be directly measured experimentally, their presence is inferred from NMR spectroscopic data, particularly through the chemical shifts (δ) of nuclei in different chemical environments. Aromatic systems exhibit characteristic ^1^H NMR chemical shifts that differ significantly from those of nonaromatic compounds [117], arising from diatropic (clockwise) ring currents. External protons in aromatic rings typically resonate downfield, at higher chemical shifts (lower field), generally in the range of 6.5–9.0 ppm. In contrast, internal protons experience shielding effects and appear upfield at lower chemical shifts, as observed for the hydrogen atoms highlighted in magenta in the aromatic systems shown in Figure 7b–d, with δ values ranging from −8.17 to −0.50 ppm. By contrast, antiaromatic systems exhibit paratropic (counterclockwise) ring currents, which induce a magnetic field that opposes B_0_ outside the ring while reinforcing it at its center. Consequently, the extent of shielding and deshielding can be used as a computational or experimental indicator of aromatic character.

Aromaticity is inferred from several physical and electronic manifestations, and no single descriptor provides a universal measure of aromatic character. Table 1 summarizes the physical basis, main strengths, limitations, and most suitable applications of commonly used structural, energetic, electronic, and magnetic aromaticity descriptors.

**TABLE 1:** Comparison of commonly used aromaticity descriptors according to their physical basis, strengths, limitations, and most suitable applications. Adapted and expanded from Ref [118].

Descriptor	Criterion and Physical Quantity	Strengths	Limitations	Uses
HOMA /HOMER	Structural: bond‐length equalization relative to reference distances. HOMER uses parameters developed for the lowest triplet state.	Applicable to experimental and calculated structures; simple to evaluate [119]; useful for comparing alternative conjugated pathways. HOMER extends the approach to selected T_1_ states.	Requires parameters for each bond type and may be affected by strain or other geometric distortions. HOMER is currently parameterized for C─C, C─N, N─N, and C─O bonds in T_1_.	Conventional organic rings and related molecular series.
ASE	Energetic: stabilization relative to suitable nonaromatic reference systems or reactions.	Directly evaluates the energetic stabilization associated with aromaticity. It can differentiate between aromatic, nonaromatic and antiaromatic, and it can be derived from experimental or calculated energies.	Strongly dependent on the choice of reference, which is often unclear for metallaaromatic compounds, transition states, and excited states. Aromatic stabilization can be difficult to separate from strain, hyperconjugation, and other effects. Conventional ASE does not provide α/β or σ/π/δ decomposition.	Small and structurally related ground‐state systems for which balanced reference reactions can be defined.
FLU	Electronic: uniformity of electron sharing between adjacent atoms relative to aromatic references	Electron‐density based. The FLU values can be separated into α and β spin components.	Standard FLU requires bond‐specific reference values. It requires caution for excited states. Depends on the selected pathway and atomic partition. Does not clearly distinguish nonaromaticity from antiaromaticity.	Local aromaticity and preferred pathways in rings, macrocycles, and nanographenes
I_ring_ and MCI	Electronic: both measure multicenter electron sharing. I_ring_ is calculated along a specified cyclic connectivity, while MCI is averaged over all permutations of the atoms in a selected ring.	Electron‐density based. Reference‐free. Spin and orbital contributions can be separated. Applicable to open‐shell and correlated wavefunctions. Hilbert‐space implementations avoid real‐space numerical integration [120].	Both are size and partition dependent and do not clearly distinguish nonaromaticity from antiaromaticity. MCI has factorial computational scaling; normalized forms reduce, but do not eliminate, size dependence.	I_ring_ for selected conjugated pathways and MCI for small‐ and medium‐sized rings (<14 atoms), polycyclic compounds, and metallaaromatic systems.
AV1245 and AV_min_	Electronic: four‐center MCI evaluated in consecutive five‐atom fragments. AV1245 gives the average along the pathway, whereas AV_min_ gives the smallest absolute contribution.	Electron‐density based. Reference‐free, low computational cost that increases linearly with ring size, avoids the numerical accumulation and factorial cost of full‐ring MCI.	Cannot be calculated for rings smaller than six atoms, depends on the selected pathway and atomic partition. Do not clearly distinguish antiaromatic from nonaromatic systems.	Large rings, macrocycles, porphyrinoids, Möbius structures, nanographenes, and comparison of various conjugated pathways.
EDDB	Electronic: fraction of the one‐electron density involved in conjugated multicenter bonding. Its integral gives the number of electrons assigned to delocalized bonds.	Quantitative and visually interpretable. Global, fragment, pathway, and ring analyses are available. Contributions can be separated into α/β or σ/π/δ components.	Global EDDB includes conjugated bonding throughout the selected system and is not restricted to cyclic delocalization. Pathway analysis and the Cyclic Delocalization Index depend on the selected atomic sequence.	Large conjugated systems, macrocycles, fused structures, and 3D aromaticity. Suitable for assessing global and local aromaticity.
NICS	Magnetic: negative magnetic shielding or shielding‐tensor component at selected points	Easy to calculate. Modern versions allow scans and α/β or σ/π/δ decomposition. It distinguishes diatropic and paratropic responses.	Depends on probe position and tensor component; may contain σ, core‐electron, local, and neighbouring‐ring contributions; affected by ring size and small orbital‐energy gaps; may produce aromatic‐like responses in intrinsically nonaromatic systems.	Simple rings and closely related series, preferably using NICS(1)_zz_, π‐only components, or NICS scans rather than isolated NICS(0) values.
Ring Currents	Magnetic: induced current density and integrated current strength	Provides the direction, intensity, and pathway of the magnetic response. Distinguishes local and global circuits, diatropic and paratropic currents, and it is more informative than NICS values.	Influenced by ring size and occupied–virtual orbital‐energy gaps; strong currents do not directly measure energetic stabilization or intrinsic electron delocalization. The magnetic‐field orientation and integration plane require careful selection, particularly for nonplanar systems.	Polycyclic systems, macrocycles, porphyrinoids, and systems with competing current pathways

These descriptors probe different molecular properties and should therefore be considered complementary rather than interchangeable. Magnetic indices, by construction, probe the response of the electron density to an external magnetic field, whereas electronic, energetic, and structural indices evaluate intrinsic features of molecular systems associated with aromaticity. The strength of an induced ring current depends on virtual transitions between occupied and unoccupied molecular orbitals [121]. These contributions are stronger when the orbitals have suitable symmetry and are close in energy. As a result, a molecule may display a strong diatropic or paratropic ring current even when its intrinsic electron delocalization and energetic stabilization or destabilization are weak. For a molecule to be aromatic, it must inherently exhibit characteristic features such as electron delocalization, specific structural organization, and enhanced stability relative to nonaromatic analogues. Nevertheless, magnetic indices often correlate well with other aromaticity measures and remain valuable tools for characterizing aromatic systems [101, 122, 123, 124, 125]. Their interpretation, however, continues to be debated, and several studies have reported differences between magnetic, electronic, and energetic criteria [78, 96, 100, 126, 127, 128, 129, 130, 131, 132, 133, 134, 135].

In some cases, magnetic measures may indicate a stronger aromatic or antiaromatic character relative to energetic, electronic, or geometric approaches. For instance, planar systems such as (N_6_H_6_)^2+^ and C_2_N_4_H_6_ exhibit ring currents comparable to benzene, yet are significantly less aromatic when evaluated using electronic criteria [96]. Similarly, as discussed at the end of the previous section, ^3^B_4_N_4_H_8_COT‐A shows only weak or negligible aromatic character according to energetic and electronic descriptors, whereas magnetic analyses suggest pronounced Baird aromaticity [98]. In addition, studies on radical ions of monocyclic hydrocarbons (including benzene, pyridine, and diazines) indicate that ring currents are not reliable descriptors for energetic stability [96]. Further evidence from expanded porphyrins [136] and porphyrin nanorings reveals even more pronounced discrepancies [94, 95], arising from the coexistence of local and global ring‐current contributions. Our own work on B_12_I_12_ clusters [137], similarly confirms that magnetic responses do not always correlate with energetic stability or other aromaticity indicators. Collectively, these studies emphasize the need for cautious interpretation of aromaticity metrics and awareness of the inherent limitations of each descriptor. In this context, the toy models recently proposed by Vanden Berghe et al. provide a valuable framework to diagnose and anticipate intrinsic NICS biases, helping to distinguish physically meaningful magnetic aromaticity trends from artifacts arising from how NICS weights different regions of the current density response [138].

In summary, magnetic indices are powerful and widely used tools in aromaticity analysis, but their interpretation remains debated because magnetic response does not always agree with energetic stability [139] or electron delocalization [140]. This has led to a conceptual distinction between “response aromaticity,” associated with magnetic behavior, and “intrinsic aromaticity,” related to electronic, energetic, and structural features [34, 141]. A comprehensive evaluation of aromaticity therefore requires the combined use of multiple descriptors, recognizing that magnetic and nonmagnetic indices arise from fundamentally different physical origins and may not always lead to consistent conclusions.

## 3 Challenges in Excited State, Macrocyclic, and Multidimensional Cases

The electronic structure of molecules in their excited states is often considerably more complex than in the ground state, posing significant challenges for the evaluation of aromaticity. Likewise, polycyclic and macrocyclic systems [142, 143, 144], which may involve multiple and sometimes extensive delocalization pathways, cannot be readily rationalized within traditional aromaticity frameworks. These difficulties are further amplified in multidimensional molecular systems, such as doubly aromatic or 3D architectures, where several interacting delocalization modes must be considered simultaneously. The present section provides an overview of the current literature addressing these issues, together with the main conceptual and methodological challenges associated with aromaticity assessment in such complex scenarios.

### 3.1 Excited‐State Aromaticity

The extension of aromaticity concepts to excited states originated from the theoretical interpretation of the Woodward–Hoffmann rules for pericyclic reactions [145, 146, 147] and was later formalized through Baird's rule [80]. In recent years, excited‐state aromaticity (ESA) has become increasingly important for rationalizing both experimental observations and computational results [32, 141, 148]. However, its application is not always straightforward, and in some cases Baird's rule has been misapplied, leading to incorrect interpretations [149, 150, 151]. In contrast to Hückel's rule, Baird's rule predicts that cyclic conjugated systems with 4n π‐electrons are aromatic in the lowest π, π* excited states (S_1_ and T_1_), whereas systems with 4n + 2 π‐electrons become antiaromatic (Figure 8). This description is primarily valid for monocyclic systems and assumes a localized ππ* excitation within the ring [152]. It is typically associated with single‐electron excitations, most commonly HOMO → LUMO transitions [153]. As a consequence, its applicability is limited in cases involving heteroaromatic systems with nπ* states [151], as well as in systems where the excitation involves Rydberg, charge‐transfer (e.g., MLCT), or other nonlocalized electronic transitions. In addition, the rule does not hold when the excitation significantly involves orbitals outside the cyclic π‐system.

**FIGURE 8:**
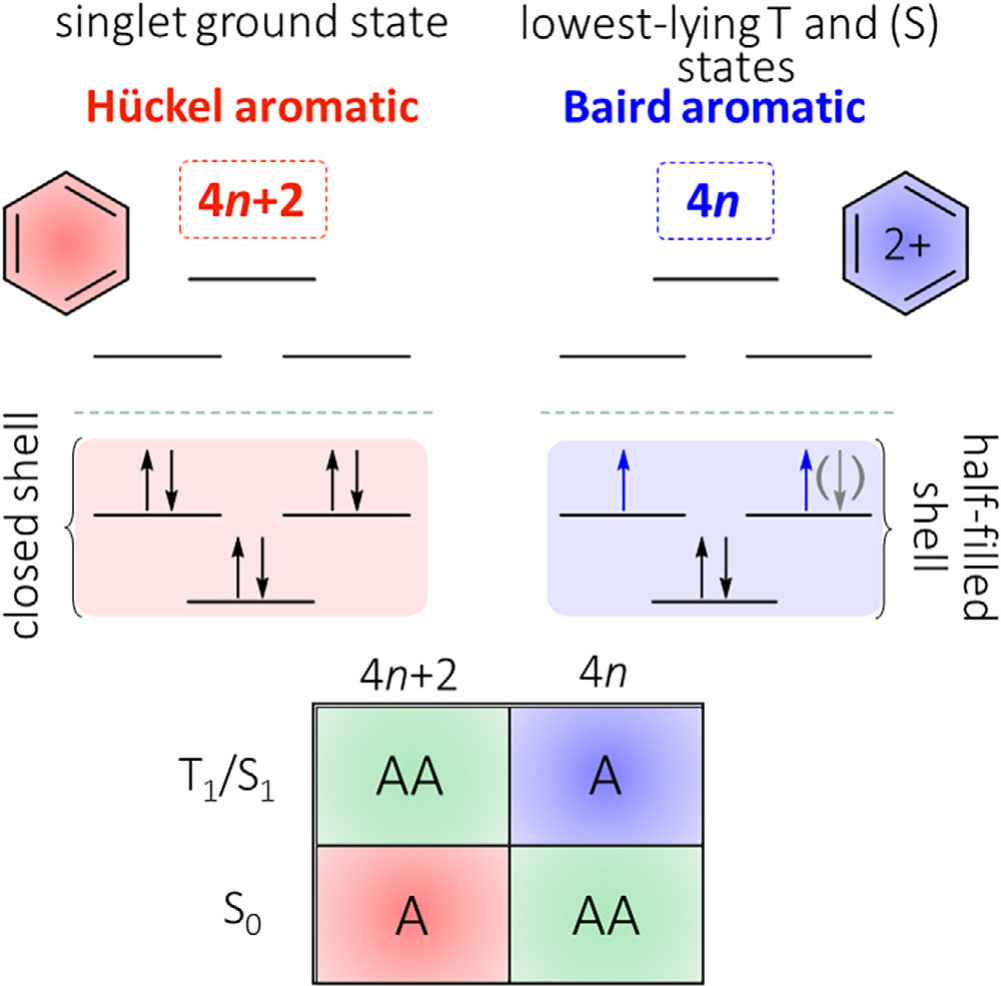
Hückel and Baird aromaticity rules illustrated through the distribution of π‐electrons in molecular orbitals: S_0_ state of benzene (left) and the low‐lying T (or S, shown in grey parentheses) excited state of the benzene dication (right). Here, “A” denotes aromaticity and “AA” denotes antiaromaticity.

The validity of the 4n/4n + 2 electron‐counting picture also diminishes with increasing ring size, which limits its applicability in large macrocycles [154, 155, 156]. Nevertheless, modified or extended formulations, often combined with Clar's rule, have successfully been applied in selected macrocyclic systems [86, 155, 157].

In certain cases, excited‐state aromaticity cannot be described using Baird's rule at all, but instead must be interpreted through a Hückel‐type framework. This occurs when excitation alters the effective π‐electron count of the ring, typically via charge transfer between the ring and exocyclic substituents. Such situations are particularly relevant in systems that evolve from nonaromatic S_0_ states to aromatic excited states (S or T). Representative examples include the T_1_ state of Cibalackrot‐type compounds [158] (Figure 9a) and the bright S_2_ state of a 6‐aminosubstituted fulvene‐based molecular motor [159], as well as quinoid systems exhibiting mixed Hückel–Baird character in S_1_ and T_1_ states [149, 150]. On the other hand, the design of principles for promoting excited‐state Hückel/Baird aromaticity was found out in symmetrically substituted pro‐aromatic quinoidal compounds [150]. Computational results identified two key strategies to promote Baird aromatic character of the T_1_ state in these compounds: (i) small anionic conjugated rings with electron‐donating substituents and electron‐withdrawing exocyclic groups, or (ii) electron‐deficient conjugated rings bearing exocyclic electron‐donor substituents (Figure 9b). Across all these cases, a central factor governing aromaticity is the change in electron count within the cyclic framework, whether through π–π*, n–π*, or charge‐transfer excitations, or through redistribution between ring‐centered and exocyclic orbitals. This determines whether Hückel‐ or Baird‐type descriptions are appropriate. To provide a more general framework, Mandado et al. [160]. proposed a spin‐resolved interpretation of Hückel's rule for open‐shell systems, separating α and β spin contributions, as illustrated in Figure 9c.

**FIGURE 9:**
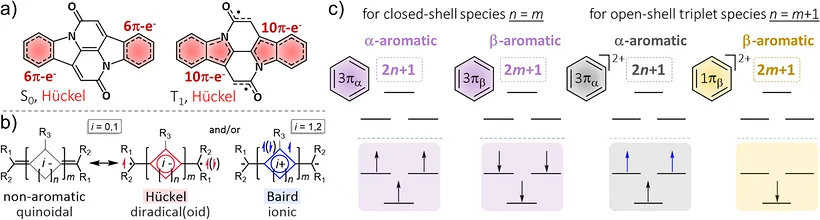
a) Cibalackrot‐type molecule in the S_0_ and T_1_ state, b) Quinoidal, radical, and ionic forms of pro‐aromatic quinoidal molecules. c) Mandado's 2n + 1 rule for separate spin channels illustrated through the distribution of π‐electrons in molecular orbitals: S_0_ state of benzene (left) and T_1_ state of the benzene dication (right).

This approach, known as Mandado's 2n + 1 rule for separate spins [160], enables a more consistent interpretation of aromaticity in radical systems. For example, in the S_0_ state of benzene and the T_1_ state of benzene dication, the system can be decomposed into two independent Hückel‐aromatic subsystems, each satisfying a 2n + 1 electron count in the α and β spin manifolds. This framework is particularly useful for understanding the ^1^n,π* and ^3^n,π* states of 6π‐electron heteroaromatics [151]. The excitation of an electron from an in‐plane n orbital to a π* orbital leaves the π system with four electrons of one spin and three of the other, making the four‐electron component antiaromatic and the three‐electron component aromatic according to Mandado's rule. These contributions rarely cancel exactly, so the overall state may retain an aromatic or antiaromatic residual. For instance, in the ^3^n,π* state of pyrazine the total MCI is 70% of its S_0_ value, well above the 50% expected for the exact cancellation. This aromatic residual mainly results from a more uniform π_β_‐electron distribution. More broadly, Mandado's framework also provides a useful tool for distinguishing between Hückel‐ and Baird‐type aromatic cycles in computational studies [149, 150, 158]. A similar rule to that of Mandado's rule, derived by Valiev et al., tells that molecules are aromatic (antiaromatic) when having an odd (even) number of doubly and singly occupied π‐orbitals [161]. This rule suggests that the aromaticity of the π,π* triplet and singlet states is the same. Finally, evaluating (anti)aromaticity in excited states and open‐shell systems remains inherently challenging. Standard electronic structure methods such as density functional theory (DFT) are often insufficient, particularly for singlet excited states, where multireference character may be significant. Linear‐response time‐dependent DFT (TD‐DFT) is useful for states dominated by single excitations, but may become unreliable for states with double‐excitation or multireference character. In such cases, multiconfigurational approaches such as CASSCF, complemented when necessary by CASPT2 to account for dynamic correlation, are more appropriate, albeit at substantially higher computational cost and interpretational complexity. When several states are close in energy, state mixing and changes in root ordering should be monitored by examining the character and dominant configurations of each state rather than relying only on state labels. For unrestricted open‐shell calculations, ⟨S^2^⟩ has to be checked, since spin contamination may affect the electron density. These limitations also affect aromaticity descriptors: methods such as NICS may require multiconfigurational treatments [84], while electronic or geometric indices such as FLU or conventional HOMA may lose validity due to the absence of appropriate reference systems. Recent developments, such as HOMER [162], a reparameterized form of HOMA for excited states, partly address this limitation for T_1_‐state aromaticity, although their applicability remains system‐ and state‐dependent. In addition, no density functional is universally optimal for aromaticity calculations. Particularly for macrocycles and porphyrinoids, representative systems should be benchmarked against experimental data or higher‐level methods when possible, and the robustness of the conclusions should be tested using functionals with different amounts and ranges of exact exchange, since delocalization error may affect geometries, electron sharing, and magnetic responses.

From an experimental perspective, probing excited‐state aromaticity is equally demanding. NMR spectroscopy is generally inaccessible in excited states, and transient spectroscopic techniques such as transient absorption spectroscopy provide only energy‐gap information rather than direct structural or electronic descriptors [163, 164, 165]. Structural methods such as X‐ray diffraction are also limited to ground‐state geometries, preventing direct observation of excitation‐induced structural changes. These constraints highlight the importance of computational approaches, while also emphasizing the need for careful interpretation to avoid misleading conclusions.

### 3.2 Aromaticity in Macrocyclic and Polycyclic Compounds

Understanding the electronic structure of macrocyclic and polycyclic systems poses unique challenges due to factors such as torsional strain, conformational flexibility, and the incorporation of heteroatoms. The study of aromaticity in these species, which are characterized by large cyclic frameworks and/or multiple fused or interconnected rings, requires consideration of both macrocyclic and global aromaticity concepts. Macrocyclic aromaticity is associated with specific pathways of π‐electron circulation along a large cyclic framework, whereas global aromaticity refers to electron delocalization extending over the entire molecular architecture through the superposition of multiple conjugation pathways. In addition, these systems may display either extended macrocyclic delocalization or aromatic character restricted to local subunits, reflecting the intricate interplay between local, macrocyclic, and global contributions [60, 166, 167].

To distinguish between these regimes, local and macrocyclic aromaticity are typically evaluated using computational indices that rely on predefined atomic circuits. In contrast, global aromaticity is more appropriately assessed through visualization‐based approaches, such as electron density analyses or magnetic current density maps obtained from response‐theory methods [168]. These distinct manifestations of aromaticity are commonly observed in porphyrin systems, where locally aromatic pyrrole units coexist with extended π‐conjugated macrocyclic pathways (Figure 10a), both of which contribute to the overall aromatic character [169]. Figure 10c illustrates that electronic and magnetic analyses may provide different pictures: the ACID plot indicates a continuous global diatropic ring current around the expanded‐porphyrin perimeter, whereas the discontinuous EDDB_G_ isosurface reveals cyclic electron delocalization confined to the pyrrolic subunits. A similar situation was anticipated in more complex architectures such as porphyrin nanorings (Figure 10b) [59], where local aromaticity is retained within individual porphyrin subunits, while potential global aromaticity would arise from an extended electron pathway along the full perimeter of the macrocycle. However, the existence of such global aromaticity has not been confirmed by electronic aromaticity descriptors. Moreover, its apparent detection using magnetic criteria [59, 170] has been shown to be highly sensitive to the choice of density functional approximation, as demonstrated by Casademont‐Reig et al. [95, 171].

**FIGURE 10:**
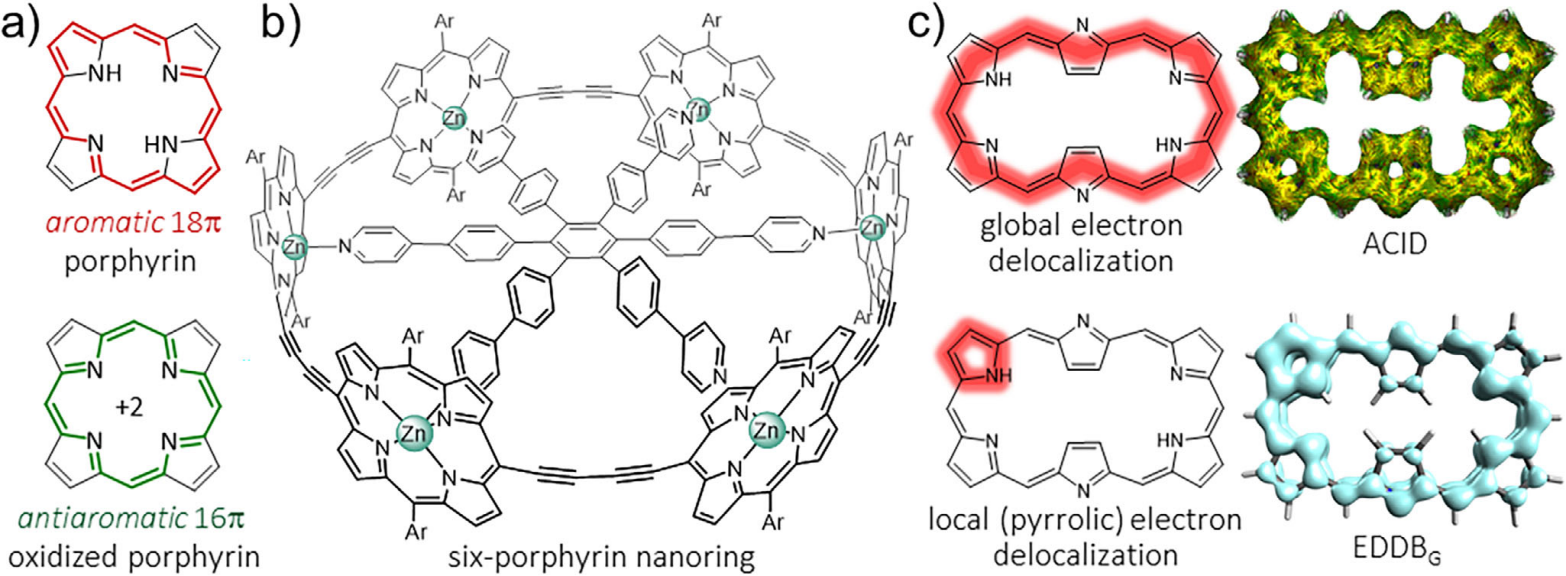
(a) Structure of porphyrin and oxidized porphyrin, with the conjugation pathway indicated according to the annulene model; aromatic and antiaromatic circuits are highlighted in red and green, respectively. Figure adapted from Ref. [172]. (b) Six‐porphyrin nanoring with aryl substituents (Ar = 3,5‐bis(trihexylsilyl)phenyl). Figure adapted from Ref. [94]. (c) A 26π electron Hückel‐aromatic expanded porphyrin illustrating the distinction between global macrocyclic and local pyrrolic aromaticity. The ACID plot shows a continuous global diatropic ring current without clear interruptions, whereas EDDB_G_ isosurface at 0.02 a.u. is discontinuous and reveals cyclic electron delocalization only within the pyrrolic subunit. Aromaticity analyses in (c) were done at CAM‐B3LYP/cc‐pVTZ level of theory.

To rationalize aromaticity in these systems, empirical rules provide a systematic framework for predicting and interpreting their aromatic behavior. The Hückel rule, originally formulated for planar monocyclic systems such as annulenes, has since been extended and complemented by additional criteria to accommodate more complex macrocyclic architectures. For instance, the parent porphyrin is often described as an 18 π‐electron aromatic system analogous to [18]annulene, thereby satisfying the Hückel rule [173]. However, such a direct comparison is not straightforward, as many macrocycles exhibit significant conformational flexibility that leads to nonplanar geometries.

This is evident in systems such as porphyrin derivatives, including p‐benziporphyrins and other Möbius‐type expanded porphyrins, as well as subphthalocyanines. The former class is associated with Möbius aromaticity [174, 175], a phenomenon observed in large [n]annulenes and expanded porphyrins that can adopt a 180° phase inversion in their π system (topology panel in Figure 6 and Figure 11a), thereby conforming to Möbius symmetry in both the molecular framework and the π‐molecular orbitals. In such cases, the extended ring size enables sufficient π‐overlap, and in closed‐shell configurations these systems can become aromatic with 4n π‐electrons.

**FIGURE 11:**
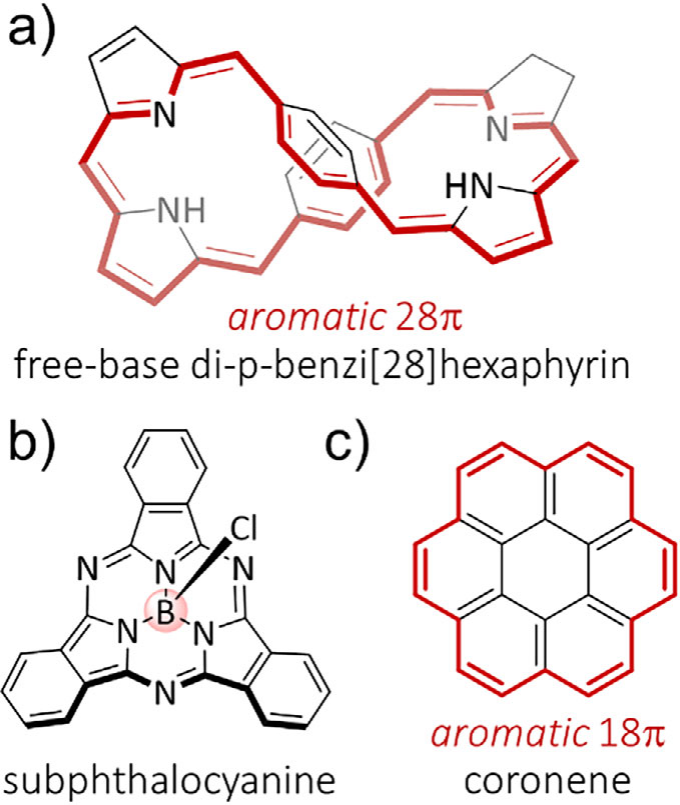
Structures of (a) free‐base di‐p‐benzi[28]hexaphyrin (Möbius aromatic), with the conjugation pathway highlighted in red; (b) boron subphthalocyanine chloride; and (c) coronene, where the molecular perimeter corresponding to the aromatic circuit is underlined in red.

Subphthalocyanines (Figure 11b), in contrast, consist of three diiminoisoindole units N‐fused around a central boron atom. This coordination enforces a nonplanar, bowl‐shaped geometry driven by both metal coordination constraints and substantial ring strain [176, 177]. These examples illustrate the tendency of large cyclic systems to adopt nonplanar conformations, thereby introducing an additional layer of complexity into aromaticity analysis.

Moreover, it has been consistently observed that the extent of aromatic stabilization tends to decrease with increasing ring size in annulenes and related macrocycles [156, 178, 179, 180]. This size dependence complicates the interpretation of aromaticity descriptors, particularly because meaningful distinctions become more difficult when the magnitude of aromatic character is small. In turn, this trend underscores the increasing difficulty of identifying and characterizing global aromaticity in larger molecular systems, where electron delocalization is often weakened or distributed across competing pathways.

Additional complications arise in the analysis of polycyclic aromatic hydrocarbons (PAHs), such as coronene (Figure 11c), and related nanographene systems composed of fused aromatic rings. Although PAHs are not strictly macrocycles, they often contain extended aromatic circuits along their perimeters or within internal subregions. These extended delocalization pathways introduce challenges in aromaticity assessment that are closely analogous to those encountered in macrocyclic systems.

In the case of coronene, a straightforward application of Hückel's rule based on the total π‐electron count (24 π‐electrons) would suggest antiaromatic character. However, coronene is experimentally aromatic [181], revealing the limitations of such a direct electron‐counting approach. This inconsistency has motivated alternative descriptions, such as the Platt perimeter model and Clar's π‐sextet rule [182, 183, 184]. Platt's model treats the outer π‐system as an annulene‐like perimeter to which Hückel's rule can be applied, effectively yielding an 18 π‐electron aromatic circuit when the inner framework is considered a perturbation. In contrast, Clar's model provides a more chemically intuitive picture by describing aromaticity in terms of π‐sextets: localized six‐electron aromatic units within hexagonal rings. According to Clar, the most stable PAH structures are those that maximize the number of disjoint π‐sextets, indicating that aromatic stabilization is not uniformly distributed but rather localized in specific regions of the molecule [185, 186].

This concept is particularly powerful for rationalizing the stability and reactivity of complex PAHs, as it highlights chemically inert regions associated with localized aromatic sextets. However, Clar's rule is formally restricted to systems composed of six‐membered rings, which has motivated extensions such as that proposed by Glidewell and Lloyd to incorporate other ring sizes [187]. These frameworks have been successfully applied to predict both structural preferences and reactivity trends in PAHs [188]. Nevertheless, in extended fused systems, aromaticity may be either cooperative or competitive between different rings, and the coexistence of locally aromatic and antiaromatic regions further complicates its overall evaluation [189].

Beyond conceptual challenges, the dimensionality and size of these systems introduce additional methodological limitations. Large PAHs often exhibit highly nonuniform electron delocalization, which directly affects the reliability of classical aromaticity indices such as HOMA and FLU, originally parameterized for small, relatively homogeneous molecules. This heterogeneity complicates quantitative comparisons and reduces the transferability of standard descriptors.

These size effects also impact both experimental and computational approaches. Experimentally, techniques such as ^1^H NMR spectroscopy may produce congested or overlapping signals due to the presence of chemically distinct but closely related proton environments across extended π‐systems. Computationally, density functional theory, while widely used, may struggle to accurately describe long‐range electron delocalization and dispersion effects that are important in large and flexible aromatic frameworks [94, 190, 191, 192]. Range‐separated hybrid functionals can improve the treatment of long‐range exchange and reduce delocalization error, although their performance remains system dependent. Moreover, selecting an appropriate level of theory is further complicated by the lack of robust reference data for benchmarking aromaticity‐related properties.

Even electronic indices considered among the most reliable, such as the MCI [193, 194], suffer from scaling limitations in large systems, often requiring approximations for practical application [22]. Moreover, MCI can fail to produce correct trends in oxidation processes [195].

From an experimental standpoint, the aromatic character of macrocyclic and polycyclic systems is best assessed through a combination of complementary techniques. X‐ray crystallography provides direct structural information, whereas atomic force microscopy, ^1^H NMR, UV–Vis spectroscopy, and related methods offer indirect but valuable insight into electronic structure, magnetic response, and delocalization patterns. Ultimately, the central challenge does not lie in the existence of aromaticity as a concept, but rather in its operational definition across different criteria. Because aromaticity is not defined by a single universal metric, different descriptors and criteria do not always lead to the same interpretation [195].

A key objective is therefore to improve the agreement between computational predictions, particularly ^1^H NMR‐derived properties, and experimental observations, going beyond methodological refinements to better reproduce realistic environmental effects in simulations. This is especially important for large, flexible systems such as nanorings, where conformational dynamics, aggregation phenomena, and vibrational contributions play a decisive role. By integrating experimental and theoretical perspectives, a more consistent and predictive description of aromaticity can be achieved, ultimately enhancing the reliability of computational chemistry in complex molecular systems.

### 3.3 Double Aromaticity

Aromaticity, traditionally associated with delocalized π‐electrons, can also emerge in all‐metal and semimetal clusters through additional types of electron delocalization, including σ‐, π‐, and φ‐aromaticity [61, 63, 65]. These different aromatic modes may coexist within a single system, giving rise to double or even triple aromaticity. However, such multifaceted aromatic behavior is comparatively rare in organic molecules, where π‐ and σ‐aromaticity dominate, and genuine examples of double aromaticity remain exceptional [77, 196, 197].

Hückel's and Baird's rules, originally formulated for π‐electron systems, can in principle be extended to σ frameworks as well, and a similar analogy applies to many of the computational indices used to quantify aromaticity. As a result, the assessment of double aromaticity inherits the same fundamental challenges encountered in general aromaticity analysis, including the absence of universal reference standards, inconsistencies between different descriptors, and the dependence on the chosen computational methodology. If not carefully addressed, these issues may lead to misleading interpretations of aromatic character.

A notable example is cyclooctadeca‐1,3,5,7,9,11,13,15,17‐nonayne (commonly known as cyclo[18]carbon, C_18_), which has been described in the literature as strongly aromatic with a proposed double Hückel in‐plane and out‐of‐plane π‐aromatic character [198, 199, 200]. However, detailed analyses indicate that in C_18_ the π‐electrons are largely localized within the triple bonds [199, 201], and the pronounced bond‐length alternation (BLA) is more consistent with weak or negligible delocalization. This suggests that, despite formal classifications, its true aromatic character is likely limited [202, 203, 204], highlighting once again the need for caution when assigning complex aromaticity labels. These conflicting assignments mainly reflect the different physical quantities evaluated by the descriptors. Magnetic indices may indicate a diatropic response even when bond‐length alternation and electronic indices reveal weak intrinsic electron delocalization.

A clear example of double aromaticity in organic systems is the hexaiodobenzene dication (C_6_I_6_^2+^) [205, 206], which exhibits both Hückel‐type π‐ and σ‐aromaticity. In this system, 10 electrons contribute to σ‐aromaticity within the iodine‐based ring, while an additional six electrons sustain π‐aromaticity in the benzene framework. Related hexahalobenzene dications have also been investigated computationally as potential candidates for double aromatic behavior [207, 208, 209, 210].

In 2018, Saito et al. reported a related case in hexakis(phenylselenyl)benzene dication, in which both π‐ and σ‐aromatic contributions were identified, although the σ‐aromatic character was found to be relatively weak [211]. In addition, Fowler and Havenith explored a series of larger 8‐membered ring systems, C_8_I_8_^q^ (q = −2 to +4), and showed that strong iodine–iodine steric repulsion induces highly puckered geometries that ultimately suppress both π‐ and σ‐aromaticity [212].

Taken together, these studies highlight that confirmed examples of double aromaticity in organic systems remain scarce. This scarcity indicates that the main challenge does not lie in the characterization of such systems, but rather in their rational design and realization, which requires a delicate balance between electronic delocalization and structural constraints.

### 3.4 Three‐Dimensional Aromaticity

The discovery of the *closo* borane [B_10_H_10_]^2−^ [72], together with subsequent studies on its electronic structure and related derivatives [73, 74, 213, 214], led to the extension of the aromaticity concept from 2D planar systems to 3D architectures. The exceptional stability observed in certain [B_n_H_n_]^2−^ clusters has been attributed to this form of 3D aromaticity [215]. However, the traditional rules developed for planar organic annulenes are not directly transferable to these polyhedral systems, which motivated the development of alternative electron‐counting schemes.

Wade introduced a classification based on valence electron counting [216, 217], in which *closo* boranes are considered aromatic when they possess 2n + 2 skeletal valence electron pairs, excluding those associated with B–H bonds, where n corresponds to the number of vertices of the polyhedral framework (represented as purple spheres in Figure 12). In parallel, Mingos proposed a related formulation that explicitly includes all valence electrons, including those from external B–H bonds, leading to an equivalent 4n + 2 electron‐counting rule [218, 219]. These two approaches are now commonly unified under the Wade–Mingos rule.

**FIGURE 12:**
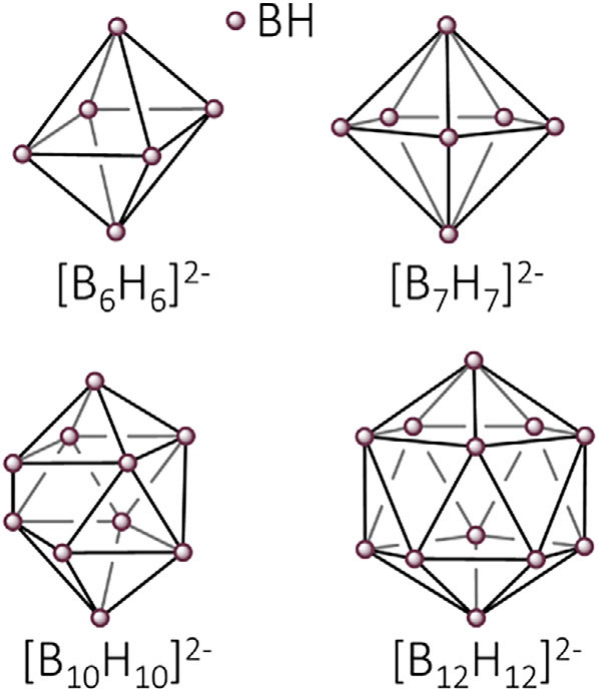
Molecular structures of B_n_H_n_^2−^ *closo* boranes with n = 6, 7, 10, and 12.

Although both Hückel's and Wade–Mingos’ rules involve a 4n + 2‐type electron count, the definition of n differs fundamentally between them. In Hückel's rule, n is a natural number and 4n + 2 refers to the number of π‐electrons in a cyclic, planar conjugated system, whereas in the Wade–Mingos framework, n corresponds to the number of boron vertices in a polyhedral cluster, with 4n + 2 representing the total skeletal valence electron count. In this sense, the two rules have been interpreted by some authors as “two sides of the same coin” [220, 221, 222], reflecting a deeper conceptual analogy between 2D and 3D aromaticity.

Beyond electron‐counting arguments, the aromatic character of boron clusters has also been supported by quantum‐chemical analyses, starting from Aihara's resonance energy calculations in 1978 [215], and further corroborated through molecular orbital interpretations and occupancy patterns, as well as numerous subsequent computational studies [222, 223].

The concept of 3D aromaticity extends beyond the cage‐like stabilization observed in icosahedral boron clusters. Additional recognized manifestations include spherical, cylindrical, octahedral, tetrahedral, and cubic aromaticity [31]. Among these, spherical aromaticity is particularly prominent and was originally introduced to rationalize the exceptional stability of fullerenes. These quasi‐spherical, π‐conjugated polycyclic structures represent archetypal examples of 3D aromatic systems [224].

For such species, Hirsch's rule, expressed as 2(n + 1)^2^ [225, 226], provides a useful electron‐counting framework by describing the π‐electron system as a spherical electron gas delocalized over the surface of a sphere, in analogy with Hückel's rule for planar aromatic molecules. This perspective highlights a broader generalization of aromaticity concepts across molecular topologies of different dimensionalities [221].

The study of 3D aromaticity thus challenges classical models developed for planar systems, offering important insights into the stability and reactivity of emerging molecular architectures with potential applications in materials science and nanochemistry. However, the inherent complexity of 3D structures also introduces significant challenges in their characterization. In many cases, simple electron‐counting rules are insufficient, requiring more detailed analyses of the underlying electronic structure.

As in previous contexts, the ambiguity of the aromaticity concept can lead to misinterpretations. A representative example is the initial assignment of 3D aromaticity to a triphenylamine‐based quinoidal bithiophene molecular cage [227], which was later re‐evaluated by El Bakouri et al., demonstrating that the system instead exhibits 2D aromaticity embedded within a 3D framework [76]. In such cases, electron delocalization and the associated electronic and magnetic responses remain confined to 2D pathways rather than extending comparably along all three spatial directions. This work highlights common pitfalls in the interpretation of aromaticity and establishes four essential criteria for true 3D aromaticity: (i) significant global π‐electron delocalization; (ii) a highly symmetric structure exhibiting at least triply degenerate molecular orbitals; (iii) a closed‐shell electronic configuration consistent with aromatic electron counts; and (iv) comparable electronic and magnetic responses along the three spatial (x, y, z) directions [76].

## 4 Understanding Leads to Design: Materials for Practical Applications

Chemistry plays a central role in addressing contemporary global challenges, ranging from energy storage and conversion to the development of cost‐effective pharmaceuticals. The discovery of new molecules and functional materials, such as catalysts, organic electronic components, and drugs, lies at the core of these efforts [228]. Achieving this requires a deep understanding of matter at the molecular and electronic‐structure level, which is made possible through advanced spectroscopic techniques and quantum chemical computations.

By the end of the 20th century, it was estimated that approximately two‐thirds of all known compounds were either fully aromatic or contained aromatic substructures [229]. This remarkable prevalence, together with the continued discovery of aromatic systems in recent decades, underscores the central importance of aromaticity in chemical research. In this context, two broad classes are particularly relevant: (i) the excited‐state properties of aromatic molecules, and (ii) systems with complex architectures, such as macrocycles, nanomaterials, and boron clusters, exhibiting cyclic or 3D electron delocalization.

This section discusses key aspects required to understand the practical implications of excited‐state aromatic molecules and structurally complex systems. The goal is to highlight how their intrinsic molecular features underpin a wide range of technological applications, thereby connecting theoretical concepts with real‐world uses of aromatic systems.

### 4.1 Electronic Nature of Systems in the Excited State

Solar radiation is one of the most abundant energy sources on Earth. Numerous fundamental processes, such as photosynthesis, vision, and climate regulation, depend directly on sunlight. Solar energy spans a broad spectral range, from ultraviolet (UV) to infrared (IR), and interacts with aromatic and heteroaromatic molecules present in both biological systems and synthetic materials [230]. Understanding light–matter interactions is therefore essential not only for natural processes but also for the development of technologies that efficiently harness this resource.

Effective utilization of solar radiation and other light sources requires a detailed understanding of photophysical and photochemical processes. In recent years, one major research focus has been the interaction of matter with visible and UV light, commonly referred to as electronic spectroscopy [231, 232]. In this regime, photon absorption promotes molecules from low‐energy electronic ground states (GS) to electronically excited states (ESs). Following excitation, excess energy is dissipated through radiative (emissive) and nonradiative pathways, as illustrated in Figure 13a. The accessibility of these processes is governed by selection rules derived from the transition dipole moment integral involving the initial (Ψ) and final (Ψ′) wavefunctions and the electric dipole operator ($\hat{\mu }$). Transitions are formally allowed when spin, orbital, and vibronic selection rules are satisfied; however, these rules rely on approximations (e.g., the Born–Oppenheimer separation), and nominally forbidden transitions may still occur under perturbations, albeit with reduced intensity.

**FIGURE 13:**
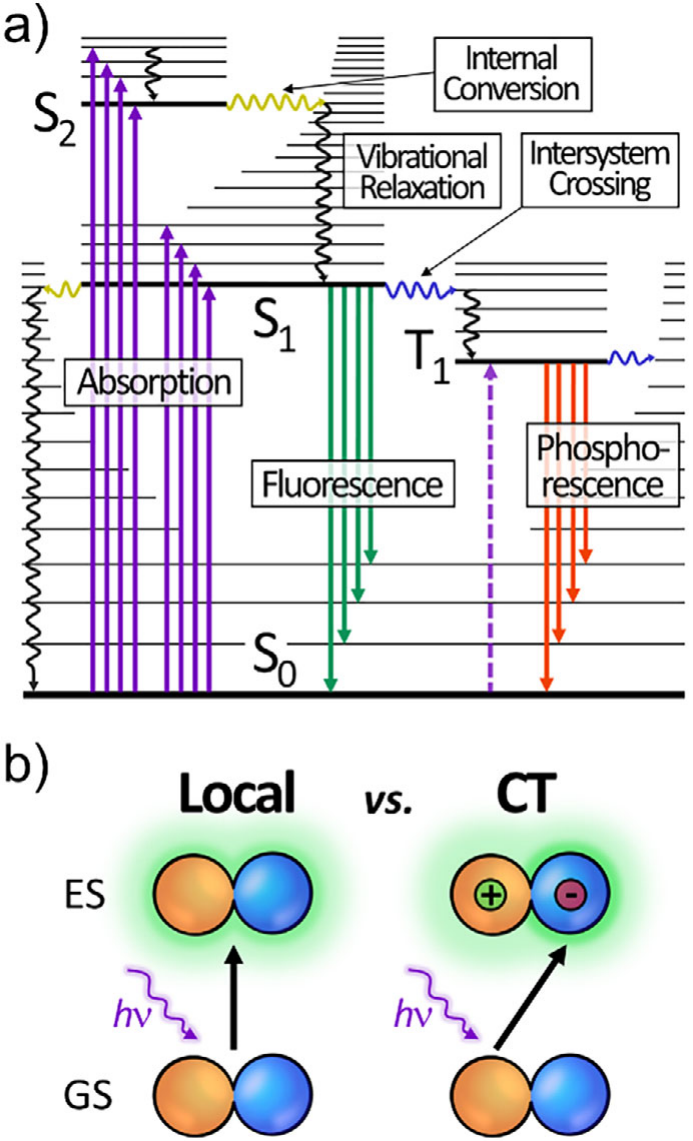
Summary of the main photophysical processes. (a) Jablonski diagram illustrating the principal relaxation pathways following photoexcitation. Straight arrows indicate radiative transitions, dashed arrows represent weak or spin‐forbidden transitions, and wavy arrows denote nonradiative processes. (b) Schematic representation of local excitation (LE) versus charge‐transfer (CT) excitation, where GS and ES denote ground and excited states, respectively.

A representative case is shown in the Jablonski diagram (Figure 13a) [233], which describes typical photophysical behavior in organic molecules. Optical excitation tends to populate the first singlet excited state (S_1_) and, to a lesser extent, higher states such as S_2_. The lowest triplet state (T_1_), which often shares a similar orbital configuration with S_1_, is typically more stable due to Hund's rule, which favors high‐spin configurations that minimize electron–electron repulsion [234]. Despite its lower energy, T_1_ is not directly accessed upon absorption because singlet‐to‐triplet transitions are spin‐forbidden. However, when competing deactivation pathways (fluorescence and internal conversion) are inefficient, population of T_1_ via intersystem crossing (ISC) can become significant. Efficient ISC requires a small energy gap between states and strong spin–orbit coupling, which enables mixing of different spin manifolds [235, 236]. Ultimately, the rates of these processes are determined by the topology of the underlying potential energy surfaces (PESs) [237], which are influenced by both intrinsic molecular properties and external perturbations.

Understanding the evolution of molecular structure in both ground and excited states is therefore essential for explaining a wide range of phenomena, including photovoltaic energy conversion [238], chemical reactivity [239, 240], photodynamic therapy activation [241, 242], photoprotection mechanisms [243], and enzymatic function [244], among others.

Focusing on a static, electronic‐structure‐based description of selected systems, and thus omitting the importance of dynamical processes, the analysis is restricted to optimized stationary points corresponding to S_0_, S_1_, and T_1_ states [245]. These represent key reference structures for understanding structure–property relationships and for guiding the rational design of functional materials. However, this approach necessarily provides only discrete “snapshots” of the full potential energy landscape, thereby simplifying the inherent complexity of real systems. A complete description would require explicit consideration of molecular and excited‐state dynamics [246, 247], as well as environmental and external‐field effects.

The analysis of excited‐state electronic structure naturally leads to the examination of how electron density redistributes upon excitation. In this task, the wavefunction (Ψ) is described within the orbital approximation, where molecular orbitals (MOs) are expressed as linear combinations of atomic orbitals (LCAO) and constructed under the Pauli exclusion principle. Accordingly, Ψ is represented either by a single Slater determinant or a multiconfigurational expansion. This framework enables direct interpretation of electronic rearrangements induced by excitation, typically involving promotion of electrons between occupied and virtual orbitals or among near‐degenerate states, leading to changes in energy, geometry, electron density, bond order, and potentially aromatic character.

Electronic excitations can be broadly classified into local excitations (LE) and charge‐transfer (CT) excitations (Figure 13b). In LE processes, electrons are promoted within the same spatial region of the molecule, resulting in relatively modest changes in charge distribution. In contrast, CT excitations involve the transfer of electron density from a donor (D) to an acceptor (A), producing a substantial redistribution of charge and often leading to pronounced changes in molecular properties and reactivity.

A variety of experimental techniques, including absorption and emission spectroscopy, transient absorption [163], time‐resolved photoelectron spectroscopy [164], and two‐photon absorption [165], provide complementary insight into excited‐state behavior. Depending on the molecular response, these methods can help distinguish between LE and CT states, as well as between different electronic configurations such as ππ* and nπ* states. Such responses are also directly relevant to optical and magnetic properties, particularly in systems sensitive to external electromagnetic fields, which are central to optoelectronic applications.

Finally, the molecular requirements for functional materials span a broad range of technologies, including organic light‐emitting diodes (OLEDs), organic photovoltaics, photocatalysis, nonlinear optical materials (NLO), photodynamic therapy (PDT), and photothermal therapy (PTT), where efficient chromophores and energy‐transfer processes are essential [248]. Additional applications include organic field‐effect transistors (OFETs), spintronics, photodetectors, and biomedical imaging, where charge transport, magnetic response, and molecular recognition become dominant factors. There are representative systems such as para‐quinodimethanes, phthalocyanines, and other macromolecular architectures. Table 2 summarizes key structure–property relationships relevant to these applications, with the overarching aim of connecting fundamental concepts such as aromaticity to experimentally observable photophysical behavior.

**TABLE 2:** Summary of requirements for organic materials as candidates for photonic and electronic applications and their technological uses.

Field	Key Requirements	Uses
OLEDs	Presence of π‐conjugated electron systems enabling semiconducting behavior via overlap of π (bonding) and π* (antibonding) orbitals; HOMO–LUMO gap typically in the range of 1–4 eV [248].	Displays for a wide range of devices (smartphones, televisions, automotive displays, etc.) and solid‐state lighting technologies.
Organic Solar Cells	Strong light‐absorbing capability covering a broad portion of the solar spectrum; suitable donor–acceptor energy level alignment; high electron affinity and chemical stability of acceptor materials; efficient charge separation and transport.	Conversion of solar energy into electricity.
Photocatalysis	Ability to undergo efficient and reversible photoinduced electron transfer processes, enabling the formation and control of reactive radical intermediates [249].	Catalysis of chemical transformations driven by light.
Nonlinear Optical Materials (NLOPs)	Donor–π–acceptor architectures promoting spatial separation of frontier molecular orbitals and efficient intramolecular charge transfer processes [250].	Optical switching, photonic devices, and molecular‐scale information storage.
Photodynamic Therapy (PDT) and Photothermal Therapy (PTT)	Strong light absorption, preferably in the near‐infrared region; high efficiency of excited‐state formation; long‐lived triplet (T_1_) states; controlled post‐irradiation stability (photostability or degradability depending on application). PDT relies on generation of reactive oxygen species [251], whereas PTT maximizes conversion of absorbed light into heat [252].	Biomedical applications including selective destruction of diseased tissues with minimal damage to healthy cells.
Organic Field‐Effect Transistors (OFETs)	π‐Conjugated small molecules or polymers with strong intermolecular packing; efficient charge transport facilitated by orbital delocalization and intermolecular electronic coupling [253].	Sensors, flexible electronics, logic circuits, memory devices, and optoelectronic components.
Spintronics	Organic semiconductors composed of light elements with weak spin–orbit coupling, leading to long spin coherence and relaxation times [254].	Magnetic memory devices (MRAM), quantum information platforms (qubits), spin‐based transistors, and light‐emitting devices.

Changes in ground‐ and excited‐state aromaticity can modulate optical gaps, charge‐transfer character, molecular polarizability, and triplet‐state accessibility and lifetime, which are relevant to OLEDs, photovoltaics, nonlinear optical materials, and photodynamic therapy, respectively.

As illustrated by numerous examples of π‐conjugated molecules, particularly aromatic systems, these structures play a central role in a wide range of applications. The unique properties imparted by the aromatic core often enable the stabilization of electronic states that would otherwise be difficult to access. In certain cases, molecules exhibit a pro‐aromatic character, meaning that while they are nonaromatic in the ground state, they acquire aromaticity upon excitation [255].

Macrocyclic architectures such as porphyrinoids and phthalocyanines further expand this versatility. Their highly adaptable frameworks make them particularly attractive scaffolds for diverse technological applications. In the following sections, these systems and their functional potential will be discussed in greater detail.

### 4.2 Pro‐Aromatic Open‐Shell Diradicaloids

In recent years, molecules with electronic structures that deviate significantly from conventional orbital ordering have attracted considerable attention [256, 257]. A notable class includes systems with very small or even negative singlet–triplet energy gaps (ΔE_S0–T_ = E_T_ − E_S0_) [60, 193, 258, 259, 260, 261, 262, 263], which in some cases lead to triplet ground states [264, 265]. In other systems, the ground state is instead an open‐shell singlet (OSS) diradicaloid, which shares an almost identical spatial electronic configuration with the triplet state and therefore appears to violate Hund's rule [266, 267, 268, 269].

The enhanced stability of the OSS relative to the triplet state can be rationalized using the two‐state model proposed by Zilberg and Haas [270], or equivalently through the concept of double spin polarization (DSP) [271, 272, 273]. These frameworks suggest that, for specific geometries, the OSS can be described as a combination of resonance structures that are inaccessible in the triplet state. In the triplet configuration, Pauli exclusion enforces parallel spins, preventing electrons from occupying the same spatial region and thereby excluding certain stabilizing resonance contributions.

In contrast, the singlet state allows electron pairing and delocalization processes that involve both spin channels. Electrons from the HOMO can delocalize into vacancies in the singly occupied molecular orbitals (SOMOs), leading to two stabilizing π‐delocalization pathways: one involving α‐electrons and another involving β‐electrons. This dual stabilization contrasts with the triplet case, where only a single stabilization pathway is available. As a result, the OSS can be energetically favored in many Kekulé‐type diradicals [274].

Systems such as heptazine derivatives have further highlighted apparent violations of Hund's rule in excited states [275, 276, 277, 278, 279]. These compounds typically possess a closed‐shell ground state, but exhibit inverted singlet–triplet energy ordering in low‐lying excited states, particularly between S_1_ and T_1_ (ΔE_S–T_ = E_S1_ – E_T1_). In contrast to the usual case, where T_1_ lies below S_1_, certain systems show the opposite ordering when both states share similar orbital configurations.

In conventional photophysical scenarios, efficient population of the triplet state can reduce fluorescence efficiency by diverting excited‐state population away from radiative S_1_ decay. However, in systems with inverted ΔE_S–T_, this limitation is mitigated, making them promising candidates for applications such as OLEDs, photovoltaics, photodynamic therapy, and fluorescence‐based imaging and sensing.

Nevertheless, the existence of inverted singlet–triplet gaps remains debated, with high‐level ab initio studies (e.g., CCSD(T)/cc‐pVTZ) challenging earlier predictions [280]. Despite these controversies, stable open‐shell singlet and triplet systems remain of broad interest, both for fundamental understanding of chemical bonding and for applications in molecular magnetism [281], nonlinear optical materials (NLOPs) [282, 283, 284, 285], and spintronics [286]. In this context, particular attention can be given to Kekulé diradicaloids, also known as Chichibabin systems, para‐quinodimethanes, or pro‐aromatic quinoidal compounds. These systems exhibit aromatic–quinoidal tautomerism [219], in which the electronic structure lies between closed‐shell quinoid, open‐shell diradical benzoid, and, to a lesser extent, ionic resonance forms (Figure 14).

**FIGURE 14:**
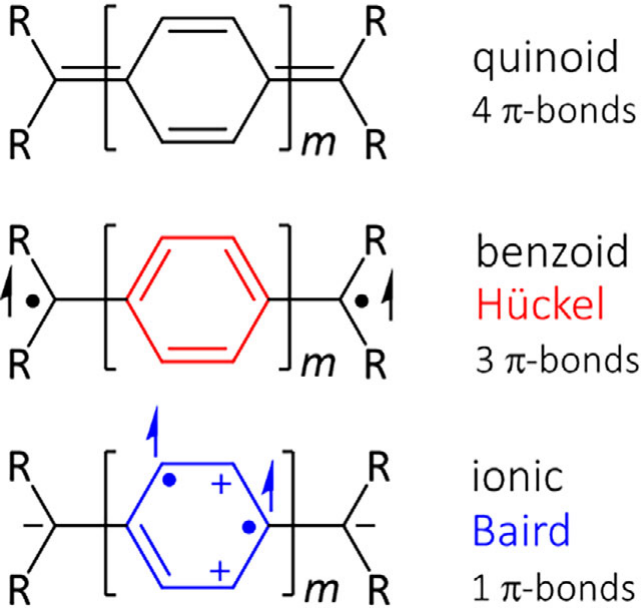
Quinoid closed‐shell, benzoid (Hückel‐type), and ionic (Baird‐type) diradical resonance forms of Chichibabin systems.

In the diradical form, two unpaired electrons may adopt either antiparallel spin alignment (open‐shell singlet) or parallel spin alignment (triplet). These species are characterized by small HOMO–LUMO gaps, which enable strong modulation of their electronic properties. However, their high reactivity often complicates experimental isolation. Structural tuning and functionalization are therefore essential strategies for controlling their properties.

A key descriptor for these systems is the diradical character, y [287, 288, 289], which quantifies the extent of diradicaloid nature. Kekulé diradicaloids span a continuum between closed‐shell (y ≈ 0) and pure diradical (y ≈ 1) limits, analogous to bond dissociation processes such as H_2_ cleavage (Figure 15).

**FIGURE 15:**
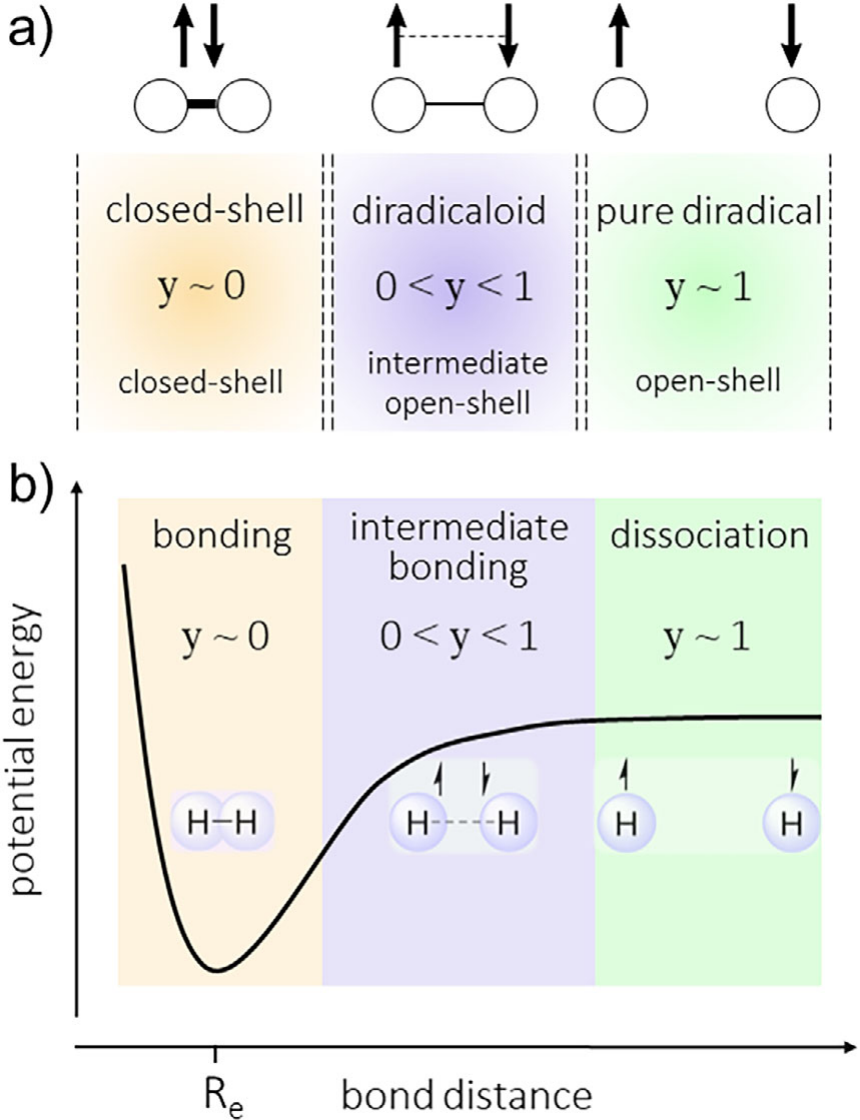
Representation of (a) singlet electronic configurations for closed‐shell, diradicaloid, and diradical species, illustrating the corresponding diradical character (y) values, with spheres representing a pair of atoms or molecular fragments; and (b) the homolytic dissociation of H_2_.

Over the past decades, significant progress has been made in stabilizing and characterizing open‐shell molecules. Structure–property relationships have been extensively analyzed using diradical character indices, ⟨S^2^⟩ values, and bonding descriptors [290]. In particular, the modulation of diradical character through structural modification has been a central theme. Key strategies include: (i) variation of terminal substituents or functional groups [291, 292, 293, 294, 295, 296], including the widely used dicyanomethylene stabilizing groups; (ii) extension of π‐conjugation length by increasing the number of repeating units (m) [255, 297, 298, 299, 300, 301, 302]. Longer conjugated chains generally stabilize the OSS state and enhance diradical character through increased aromaticity [294, 303]. Representative Kekulé diradicaloids exemplifying this trend include Thiele's (m = 1) [304], Chichibabin's (m = 2) [305], and Müller's (m = 3) hydrocarbons [306], as reflected by the progressive increase in *y* values shown in Figure 16a. Additional strategies involve (iii) chemical modification of π‐bridges or alternative substitution patterns in the central ring(s) to tune electron delocalization and steric effects [292, 307, 308, 309], and (iv) investigation of isomeric effects on chemical properties and, consequently, diradical character [310, 311]. Together, these approaches, some illustrated in Figure 16, highlight the multifaceted strategies available for designing diradical systems.

**FIGURE 16:**
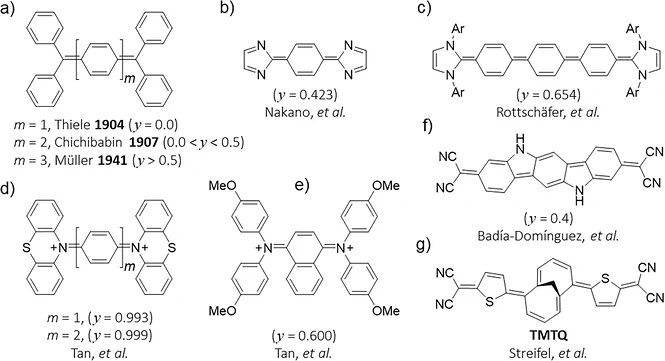
Representative para‐substituted diradicaloid systems with corresponding diradical character. (a) Thiele's [304], Chichibabin's [305], and Müller's [306] hydrocarbons. N‐heterocyclic carbene analogues of (b) Thiele's hydrocarbon, with diradical character (y) computed at the UHF/6‐31+G** level [291], and (c) Müller's hydrocarbon, where Ar = 2,6‐iPr_2_C_6_H_3_, with y evaluated at the UHF/6‐311G* level [312]. (d,e) Bis(triarylamine) dications, with y calculated at the UBH&HLYP/6‐31G* level [292]. (f) Indolocarbazole derivatives substituted with dicyanomethylene groups, with y determined at the UB3LYP/6‐31G** level [307]. (g) Covalent structure of 2,7‐dicyanomethylene‐thiophene‐1,6‐methano[10]annulene (TMTQ) [313].

In conjugated aromatic polymers, increasing chain length generally favors diradical formation over closed‐shell configurations. This trend is accompanied by a decrease in both optical and electronic band gaps. However, shorter oligomers with a single repeat unit often retain closed‐shell ground states while exhibiting accessible diradical aromatic excited states. A representative example is TMTQ, a 2,7‐dicyanomethylene‐thiophene‐substituted methano[10]annulene derivative, which exhibits a very small singlet–triplet gap (≈ 4.9 kcal/mol) and serves as a model system for studying excited‐state aromaticity.

Initially, the T_1_ state of TMTQ was described as Baird aromatic [313], but later analyses indicated that its Baird aromatic character is partial (≈10%–15%), better described as a Hückel–Baird hybrid [149]. More recently, some studies have reclassified its S_1_ state as fully Baird aromatic [314]. Given these conflicting interpretations, a detailed re‐examination of its excited‐state aromaticity was mandatory [150, 294].

Overall, aromaticity provides a powerful framework to rationalize how structural modifications, such as π‐system extension, substituent effects, and conformational changes, influence the electronic properties of para‐quinodimethane‐based systems. In particular, both singlet and triplet diradical forms often retain significant aromatic stabilization, making aromaticity a key factor in their stability. Computational characterization is therefore essential, especially for highly reactive species that are difficult to isolate experimentally, and plays a central role in guiding the rational design of functional molecular systems.

### 4.3 UV–Vis Spectra of Porphyrins and (Sub)Phthalocyanines

A prominent family of molecules exhibiting rich electronic behavior and broad relevance to excited‐state phenomena includes porphyrins and related macrocycles such as phthalocyanines and subphthalocyanines [176, 177, 315, 316]. Porphyrins are heterocyclic macrocycles composed of four pyrrole units linked through methine bridges (R_1_‐CH = R_2_), forming an extended conjugated π‐system with a central cavity that commonly hosts a metal ion. This coordination is often essential for their function. These compounds play central roles in both biological and technological contexts and display characteristic absorption features. Indeed, naturally occurring porphyrin derivatives are responsible for well‐known biological coloration, such as the red of hemoglobin and the green of chlorophyll, reflecting their involvement in oxygen transport and photosynthesis. Their diverse optical signatures arise directly from their underlying electronic structure.

A key step toward understanding and predicting the π→π* transitions in metalloporphyrin UV–Vis spectra was provided by Gouterman's four‐orbital model [317, 318]. Building on concepts from cyclic polyenes and molecular orbital theory, this model describes the frontier interactions between the occupied a_2u_ and a_1u_ orbitals and the unoccupied e_g_ orbitals. These transitions give rise to the characteristic Soret (B) and Q bands observed experimentally, as illustrated in Figure 17. Beyond reproducing spectral patterns, the model also accounts for variations in band intensity and energy, providing a practical framework to rationalize substituent effects and electronic perturbations in porphyrin derivatives.

**FIGURE 17:**
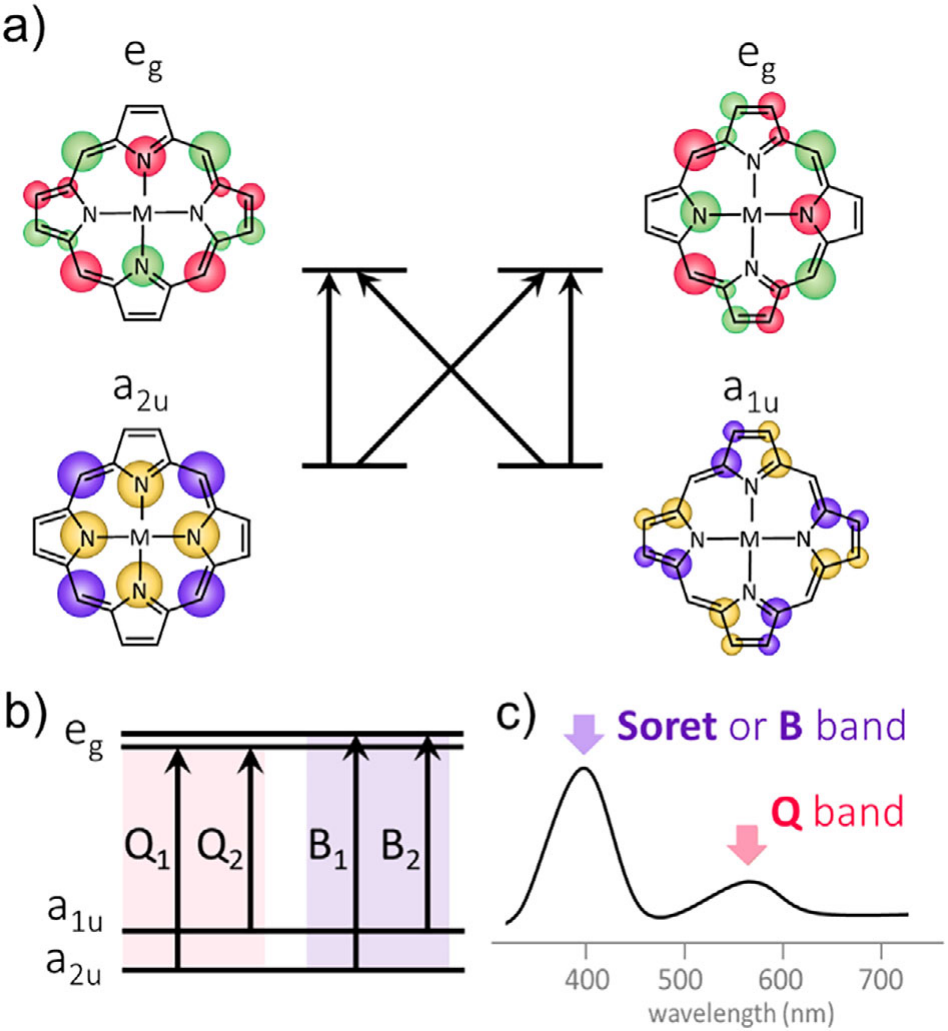
Schematic illustration of Gouterman's four‐orbital model for metalloporphyrins: (a) frontier molecular orbitals, showing the HOMOs (a_2u_ and a_1u_ symmetry) and LUMOs (e_g_ symmetry); (b) orbital energy diagram highlighting the electronic transitions responsible for the Q and B (Soret) bands; and (c) idealized UV–Vis absorption spectrum depicting the characteristic spectral features arising from these transitions.

Despite its simplicity, Gouterman's model remains a powerful interpretative tool when combined with modern spectroscopic techniques and quantum chemical calculations. Its continued relevance is reflected in the vast structural diversity of porphyrin derivatives, where subtle modifications enable fine tuning of optical responses [319]. Among related systems, phthalocyanines represent an expanded analog of porphyrins, consisting of four isoindole units connected via nitrogen atoms to form a highly planar, extensively conjugated macrocycle. This extended π‐system imparts unique electronic and optical properties, making phthalocyanines widely used in organic electronics, photovoltaics, and photodynamic therapy, among other applications [316].

Subphthalocyanines constitute a contracted structural analogue, composed of three isoindole units coordinated to a central boron fragment (B–X, where X = OH, F, Cl, etc., see Figure 11b). Unlike their planar counterparts, subphthalocyanines adopt a characteristic bowl‐shaped geometry, which strongly influences their intermolecular interactions, stacking behavior, and host–guest chemistry [320]. Despite their reduced size, they retain a highly conjugated π‐framework and represent an important example of nonplanar aromatic systems. Their curved geometry also modulates their electronic and optical responses, enabling applications in optoelectronics and medicinal chemistry [177, 321].

Collectively, porphyrins, phthalocyanines, subphthalocyanines, and their derivatives provide a versatile platform for engineering materials with tailored electronic conductivity, long‐lived excited states, and tunable optical properties [177, 316, 322, 323]. As synthetic capabilities continue to expand, establishing clear structure–property relationships becomes increasingly important for rational molecular design. In this context, UV–Vis spectroscopy serves as a particularly informative probe, offering direct insight into electronic structure and its modulation by chemical substitution and geometry.

Recent studies have highlighted connections between aromaticity and optical properties in porphyrinoid systems, including nonlinear optical responses. Examples include Hückel–Möbius aromaticity switching in expanded porphyrins with enhanced nonlinear optical properties [324, 325], studies by Woller et al. showing how (anti)aromaticity and molecular topology influence the linear and nonlinear optical behavior of porphyrinoids [172, 326], and analyses of aromatic pathways in charged expanded porphyrins [137]. More recently, Desmedt et al. found that macrocyclic aromaticity does not reliably predict absorption intensities in a diverse set of hexaphyrins. In particular, intense B bands were associated with highly planar structures, irrespective of their aromatic or antiaromatic character. They therefore cautioned against using absorption spectra alone as indicators of aromaticity [327]. Nevertheless, in specific systems, tuning aromaticity can directly modulate photophysical properties, as demonstrated for silicon(IV) phthalocyanines [328].

Motivated by these observations, and given the absence of a unified comparative study across porphyrins, phthalocyanines, subphthalocyanines, and related systems, we investigated potential correlations between UV–Vis absorption properties and aromaticity [329]. This approach combined experimental and computational analysis of absorption spectra with electronic structure and aromaticity descriptors of different conjugated pathways. Relative to porphyrin, porphyrazine, and phthalocyanine, both containing nitrogen atoms at the meso positions, exhibit reduced electron delocalization along the outer macrocyclic pathway. In particular, N‐meso substitution reduces electron delocalization along this pathway and shifts the Q band to lower energies, while benzannulation of the pyrrole units produces a similar effect by decreasing delocalization in the fragments involving β‐positions at which the benzene rings are fused. By contrast, the B‐band energy shows a tentative relationship with the aromaticity of the inner pathway rather than the outer pathway, although the correlation is weaker and therefore less informative than that observed for the Q band. Overall, the results reveal pathway‐specific links between electron delocalization and spectral trends that cannot be explained by orbital energies alone. They also show how structural changes that redistribute π‐delocalization can tune porphyrinoid optical responses, although these correlations should be restricted to closely related molecular series rather than treated as universal criteria of aromaticity.

### 4.4 Macromolecular, π,π‐Doubly and 3D Aromatic Systems

Carbon‐based materials occupy a central position across a wide range of scientific disciplines, including environmental science, energy storage, and materials chemistry. The versatility of carbon, arising from its ability to adopt sp, sp^2^, and sp^3^ hybridizations, enables an extraordinary diversity of structural motifs. Many of these architectures host stable delocalized electron networks, largely governed by π‐conjugation, and this versatility is further enhanced by the incorporation of heteroatoms. Aromaticity acts as a key stabilizing principle in many of these systems and underpins a broad spectrum of molecular discoveries, ranging from porphyrins and fullerenes to hoop‐shaped π‐systems, nanographenes, and more recently developed supramolecular architectures such as saturnene, in which a fullerene is encapsulated by a [n]cycloparaphenyleneacetylene ring [330]. These systems not only exemplify the richness of carbon chemistry but also display technologically relevant properties, including high conductivity, tunable optical responses, photoinduced electron transfer, and exceptional structural stability [331, 332, 333, 334].

An example of intriguing aromatic systems is provided by icosahedral boron clusters, including the isomeric carboranes C_2_B_10_H_12_ in both neutral and anionic forms [335]. These species are notable for their low nucleophilicity, chemical robustness, and tunable hydrophilicity. Aromaticity in carboranes and metallabis(dicarbollides) differs fundamentally from classical planar π‐systems. The greater stability and deboronation resistance of *m‐*carborane relative to *o‐*carborane arise from carbon atom positioning rather than aromaticity. Magnetic and computational analyses confirmed strong global aromaticity in *closo‐*, *nido‐*carboranes, and metallabis(dicarbollides), despite major structural differences and open geometries. Unlike planar hydrocarbons, boron clusters preserve aromaticity upon electron addition or deboronation by adapting their geometry (*closo*‐to‐*nido* transformation). These findings highlight the resilience, spherical nature, and unique electron‐counting behavior of aromaticity in boron clusters [335]. On the other hand, the possibility of achieving double aromaticity in boron clusters featuring an external iodine shell was also studied [137], with the aim of rationalizing experimental observations and broadening the conceptual framework of aromaticity in inorganic systems.

### 4.5 Porphyrin‐Related Macrocycles

Building on the previously discussed structural diversity and applications of porphyrins and phthalocyanines, we now consider specific structural modifications that give rise to a wide range of porphyrinoid derivatives. Common synthetic strategies for modifying the parent porphyrin framework include: (i) substitution of meso‐CH groups by nitrogen atoms [176, 336]; (ii) replacement of peripheral hydrogen atoms with substituents of varying electronic and steric character [337, 338], or fusion of isoindole and pyrrole units with additional ring systems [336, 339]; (iii) variation in the number of pyrrole or isoindole units to generate contracted or expanded porphyrinoids; and (iv) modification of the central coordination environment, including the incorporation of different transition metals and axial ligands in metalloporphyrins and phthalocyanines [323, 340], or the use of boron‐centered motifs in contracted analogues [176]. Each of these modifications has a pronounced effect on the physical and chemical behavior of the resulting macrocycles.

For example, peripheral substitution can be used to tune solubility [341], optical absorption profiles [339], and redox properties [342]. Likewise, the identity of the central metal ion, from transition metals such as Fe or Zn to lanthanides, imparts distinct electronic structures and reactivity patterns. This structural flexibility underpins a wide range of applications, including catalysis [343, 344] and photodynamic therapy [241, 316]. Additional diversity arises in phthalocyanine and subphthalocyanine systems, where structural changes such as isoindole replacement or metal variation strongly influence electronic properties, often enhancing light‐harvesting efficiency, charge‐transfer behavior, and chemical stability [176, 177]. In particular, the incorporation of heavier or multiple metal centers can introduce novel optical and magnetic responses, expanding their potential in advanced photonic and electronic technologies [321]. These tunable features make such macrocycles attractive platforms for applications ranging from gas sensing to organic semiconductor design.

An alternative and highly effective strategy for modulating porphyrin‐based systems involves their assembly into extended architectures such as linear tapes [345] or cyclic nanorings [58], thereby introducing new topological and electronic effects. In tape‐like structures, increased conjugation across linked porphyrin units enhances light absorption and modifies electronic structure, making these materials promising for photovoltaic and sensing applications [346]. In contrast, porphyrin nanorings, which consist of cyclic arrangements of porphyrin units, introduce a closed‐loop topology that significantly alters delocalization patterns and enables applications in artificial light‐harvesting systems [347]. These structural motifs provide valuable insight into how molecular topology governs electronic and photophysical behavior.

Finally, it should be noted that the aromaticity of porphyrinoid systems has, in some cases, been overestimated [148, 192]. Given the central role of aromaticity in determining electronic structure and associated properties, accurate and critical evaluation is essential. Mischaracterization can lead to incorrect interpretations of their chemical and physical behavior. A rigorous assessment of aromaticity is therefore necessary to ensure reliable structure–property relationships in these complex macrocyclic systems.

### 4.6 Nanographenes

Graphene is a remarkable carbon allotrope in the field of advanced materials, consisting of a single atomic layer of carbon atoms arranged in a 2D honeycomb lattice. This structure confers exceptional electronic, mechanical, and thermal properties [348]. A defining feature of graphene is its zero band‐gap character, arising from the continuous overlap between valence and conduction bands, which results in the absence of an optical band gap. While this makes graphene highly conductive, it also limits its direct use in optoelectronic applications due to the lack of light‐emission‐related properties [349].

In contrast, finite fragments of graphene—commonly referred to as graphene nanoflakes or nanographenes, typically composed of tens to hundreds of carbon atoms (and larger than ∼1 nm)—exhibit quantum confinement effects. This confinement leads to the emergence of discrete electronic states and an effective opening of the band gap [350]. As a consequence, nanographenes retain many of graphene's advantageous properties while additionally displaying photoluminescence, significantly expanding their potential applications.

Another important distinction is that pristine graphene is intrinsically achiral, whereas chirality can be introduced in nanographenes. This can be achieved via two general strategies: (i) a top‐down approach, in which chirality is introduced through supramolecular assembly or covalent attachment of chiral substituents [351, 352, 353]; or (ii) a bottom‐up synthetic strategy, where the molecular design incorporates structural defects that render the nanographene intrinsically chiral [354, 355, 356]. The latter approach is of particular interest here and is typically realized through the inclusion of nonhexagonal rings within the hexagonal carbon framework. The incorporation of five‐ or seven‐membered rings induces curvature: pentagons generate positive curvature, leading to bowl‐shaped structures such as corannulene, whereas heptagons introduce negative curvature, producing saddle‐shaped systems such as [7]circulene [357]. These topological modifications significantly broaden the structural and functional diversity of nanographenes [358].

A well‐known example of molecular chirality in such systems is [6]helicene, shown in Figure 18a. This polycyclic aromatic hydrocarbon is inherently 3D due to steric repulsion between its terminal rings, which forces the structure into a helical shape. Importantly, this chirality arises without stereogenic centers. In helical systems, the conventional R/S nomenclature is replaced by P (plus) and M (minus) descriptors, corresponding to right‐ and left‐handed helices, respectively. The configuration is assigned based on the direction of a helical path traced from one terminal ring to the other, with clockwise and counterclockwise orientations defining P and M forms.

**FIGURE 18:**
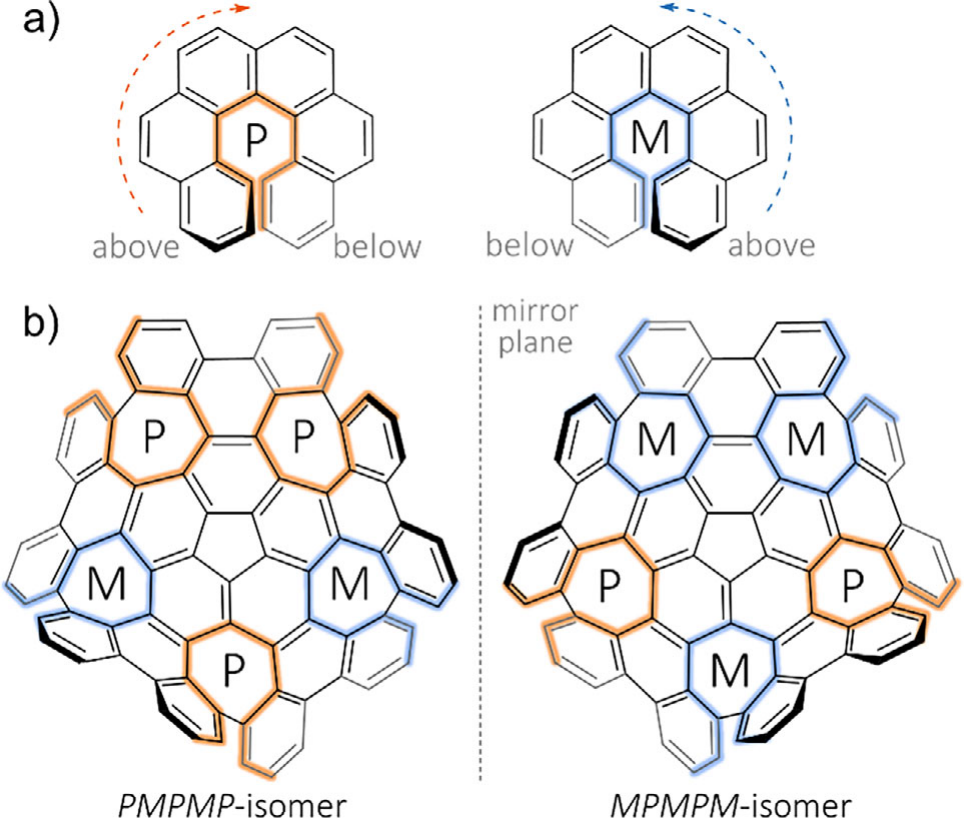
Illustration of spiral chirality through the two enantiomeric forms of (a) [6]helicene and (b) the C_80_H_30_ nanographene. The P (plus) and M (minus) descriptors denote right‐handed and left‐handed helical chirality, respectively.

The same chirality concepts extend to more complex nanographenes, such as curved C_80_H_30_ systems [354], which combine concave and convex curvature elements. These structures give rise to distinct enantiomeric forms (PMPMP‐ and MPMPM‐isomers), as illustrated in Figure 18b.

The structural variability and tunability of nanographenes enable precise control over their physical and chemical properties [358, 359], including optical response [360] and host–guest interactions with curved species such as fullerenes [361]. This versatility makes them promising platforms for supramolecular chemistry and for investigating electron‐transfer processes in donor–acceptor systems. A representative example is the work of Zhou et al. [362], where a corannulene‐based helical nanographene coordinated with sodium exhibited a switch in aromatic character upon two‐electron reduction, transitioning from aromatic to antiaromatic behavior. This highlights the sensitivity of aromaticity as a descriptor for tracking electronic changes in nanographenes.

However, the study of aromaticity in distorted nanographene architectures, which contain multiple fused and topologically diverse conjugated rings, remains particularly challenging [363]. Reliable characterization typically requires the combined use of multiple aromaticity indices. From a methodological perspective, this allows benchmarking of different aromaticity measures, while from a chemical standpoint, it is essential for understanding electronic structure and establishing robust structure–property relationships.

Overall, nanographenes provide an ideal testing ground for aromaticity concepts and their limitations. Their diverse and often highly distorted topologies not only challenge existing aromaticity models but also expand the design space of functional nanocarbon materials. This makes them key systems for both fundamental studies of aromaticity and the development of next‐generation nanoscale materials.

### 4.7 Doubly Aromatic Systems

Since the identification of the first doubly aromatic species, the 3,5‐dehydrophenyl cation (Figure 5) [77], relatively limited attention has been devoted to the discovery of organic systems capable of sustaining two orthogonal cyclically delocalized electronic networks. Although examples of σ,π‐doubly aromatic compounds have been reported in both organic and inorganic chemistry [61, 63, 65], organic representatives remain scarce and are largely restricted to doubly oxidized substituted benzenes [205, 206, 207, 208, 209, 210, 211]. A representative example is the doubly aromatic C_6_I_6_^2+^ system, in which two‐electron oxidation of neutral C_6_I_6_ creates an electronic vacancy in one of the iodine 5p orbitals. This process enables a set of resonance structures, three representative examples are shown in Figure 19, which collectively permit cyclic σ‐electron delocalization. Together with compliance with Hückel's electron‐counting rule, these resonance effects are essential for the coexistence of π‐ and σ‐aromaticity in the molecule [364], which is an unconventional aromatic regime and also an emerging frontier in organic chemistry.

**FIGURE 19:**
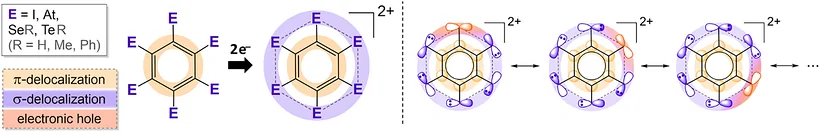
Conceptual representation of double aromaticity in dicationic hexahalo‐ and hexachalcogeno‐substituted benzenes. The left panel illustrates σ‐electron delocalization leading to simultaneous Hückel π‐ and σ‐aromaticity upon double oxidation of the neutral precursor. The right panel depicts double aromaticity in C_6_E_6_^2+^ species, where the formation of an electronic vacancy enables σ‐delocalization. Adapted from ref.137.

In these systems, substituted C_6_R_6_^2+^ compounds (R = halogen or related substituents such as chalcogens) exhibit double aromaticity, although σ‐conjugation is generally weak. This limitation arises from the relatively large distances between substituents imposed by the approximately 120° R–C–C bond angles of the six‐membered ring, which reduce effective orbital overlap. Nonetheless, these systems have inspired new conceptual strategies, including the deliberate incorporation of iodine substituents into aromatic frameworks, where reactive intermediates and iodinated oligomers can generate high‐energy in‐plane σ‐antibonding orbitals capable of substantially modifying the electronic structure [365]. More recently, the group of Radenković has extended this investigation of σ‐aromaticity generated by oxidation to model periodo‐derivatives of polycyclic (anti)aromatic hydrocarbons [366] and halogenated cycloalkanes [367], in which oxidation can induce additional σ‐electron cyclic delocalization along the macrocyclic iodine ring.

An alternative route toward double aromaticity has been explored in eight‐membered rings (8‐MRs), particularly in C_8_I_8_^q^ systems with charges q = 0, +1, +2, +4, and −2 [198, 199, 200]. However, the increased substituent overlap in 8‐MRs introduces pronounced I···I steric repulsion, leading to strongly puckered conformations that compromise effective delocalization. Motivated by the observation that substituted benzenes display measurable double aromaticity, we turned our attention to an intermediate seven‐membered ring (7‐MR) framework [364], and proposed substituted tropylium ions as promising candidates for doubly aromatic systems. This choice is supported by both structural and synthetic considerations.

Our interest in tropylium derivatives is motivated by three principal factors. First, in tropylium cations (C_7_X_7_^+^), X···X separations are intermediate between those of six‐ and eight‐membered analogues, potentially improving orbital communication while reducing steric repulsion. Second, halogen substituents can contribute to a 14 σ‐electron framework satisfying a 4n + 2 electron count, raising the possibility of an additional aromatic σ‐system. Third, oxidized species (C_7_X_7_^3+^) in their low‐lying triplet states may simultaneously exhibit Hückel aromaticity within the tropylium π‐framework and Baird aromaticity within the σ‐ring. As such, these compounds may offer a favorable balance between steric accessibility and efficient electronic delocalization. Our results show that C_7_Br_7_^3+^ in its triplet state, with an internal Hückel aromatic tropylium ring and an external incipient Baird aromatic Br_7_ ring, shows double π‐ and σ‐aromaticity. This result, however, is functional‐dependent and reveals that ^3^C_7_Br_7_^3+^ is at the borderline for onset of double aromaticity.

The tropylium cation itself is a classic example of a nonbenzenoid aromatic system, satisfying Hückel's rule through its six π‐electron conjugated framework. Investigating substituted tropylium derivatives therefore provides an appealing opportunity to expand the understanding of double aromaticity. From a theoretical perspective, these systems offer insight into electronic structure, stabilization mechanisms, and the interplay between orthogonal aromatic circuits. Beyond their relevance to fundamental quantum chemistry, such investigations may also contribute to the rational design of complex molecular architectures in which tropylium motifs already play an important synthetic role. Furthermore, studying these seven‐membered aromatic systems deepens understanding of carbocation chemistry and less conventional ring topologies, while shedding light on electronic effects such as resonance and hyperconjugation. Finally, the emergence of double aromaticity may influence measurable properties, including UV–Vis absorption, thereby opening additional opportunities for spectroscopic characterization and materials‐oriented applications.

### 4.8 3D Aromatic Systems: Boranes and Carboranes

Boranes, or boron hydrides, are generally characterized by high reactivity and limited intrinsic stability due to their electron‐deficient nature. To compensate for this deficiency, boron atoms frequently form multicenter bonding motifs, including three‐center two‐electron (3c–2e) B–B–B bonds and B–H–B bridging interactions. Despite this inherent reactivity, a subset of boron clusters—polyhedral boranes—exhibits remarkable thermodynamic and structural stability. These compounds, constructed from polyhedral frameworks composed of triangular faces, are conventionally classified into three principal families: *closo‐*, *nido‐*, and *arachno‐*boranes, which differ in geometry, electron count, and stability (Figure 20).

**FIGURE 20:**
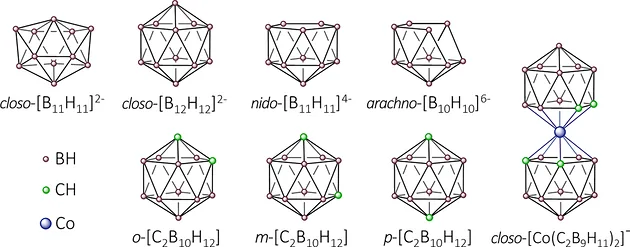
Molecular structures of *closo‐*[B_11_H_11_]^2−^ and the isoelectronic series *closo‐*[B_12_H_12_]^2−^, *nido‐*[B_11_H_11_]^4−^, and *arachno‐*[B_10_H_10_]^6−^ boron clusters (top, left to right); the *ortho*‐, *meta*‐, and *para*‐isomers of [C_2_B_10_H_12_] carboranes (bottom, left to right); and *closo‐o*‐[Co(C_2_B_9_H_11_)_2_]^−^ (right).

The *closo‐*boranes, previously introduced and shown in Figure 12, display highly symmetric, closed polyhedral geometries. A representative example is [B_12_H_12_]^2−^, which adopts a nearly regular icosahedral structure. In contrast, *nido‐*boranes possess more open architectures that can be viewed as derivatives of *closo* species generated through electron addition, hydrogen incorporation, or skeletal rearrangement. For example, adding two electrons to a *closo* cluster such as [B_11_H_11_]^2−^ without removing a vertex produces *nido‐*[B_11_H_11_]^4−^, representative of the general [B_n_H_n_]^4−^ class. Alternatively, hydrogenation may yield neutral species of formula [B_n_H_n+4_], while another pathway involves removing one vertex from a *closo* cluster containing *n* + 1 vertices without changing the electron count. *Arachno*‐boranes are generated through further skeletal opening, typically via additional electron or hydrogen incorporation, or by removal of an additional vertex relative to *nido* species.

Among these classes, *closo‐*boranes are generally the most stable and least reactive, a property closely linked to their 3D aromaticity as rationalized by the Wade–Mingos electron‐counting rules. Although these rules are widely employed in discussions of aromaticity, their principal function lies in predicting and rationalizing borane cluster geometries. Similar electron‐counting approaches, together with complementary classification schemes, extend naturally to heteroboranes and metalloboranes. Their applicability to carboranes and related derivatives is particularly valuable because such frameworks serve as versatile platforms for synthetic design, with implications spanning theoretical chemistry, catalysis, medicine, and materials science.

Carboranes arise formally from substitution of a BH^−^ unit in boranes by an isoelectronic CH fragment, producing a distinct family of polyhedral compounds closely related to boron hydrides. This substitution substantially enhances stability relative to conventional boranes because of the increased electron density introduced by carbon. Consequently, carboranes have become important motifs in the chemistry of weakly coordinating anions. Moreover, incorporation of carbon heteroatoms enables selective functionalization, considerably expanding their synthetic utility. These developments also paved the way for metallacarboranes, particularly complexes of the type [M(C_2_B_9_H_11_)_2_]^−^ (M = transition metal), commonly referred to as metallabis(dicarbollides).

The exceptional structural diversity and robustness of boranes and related species underpin their broad applicability across multiple scientific disciplines. Hydrogen‐rich *closo* boranes and their alkali‐metal derivatives, such as MB_n_H_n_ species [368], have attracted interest as superhalogens and hyperhalogens—superatomic systems capable of mimicking the chemistry of halogens and alkali metals. Likewise, the stability, aromaticity, and 3D electron delocalization of polyhedral boranes make them promising candidates for luminescent and nonlinear optical materials [369].

In medicinal chemistry, compounds such as *o*‐carborane (or carboranyl ligands) are sometimes considered phenyl bioisosteres because of similarities in lipophilicity and chemical robustness, although the analogy is not exact. Rather than serving as strict benzene replacements, carboranes provide distinctive steric and electronic environments that enable alternative molecular recognition patterns and novel pharmacological strategies. Their high thermal and chemical stability, together with tunable electronic properties, have therefore stimulated growing interest in drug design and materials development. Additionally, owing to their high gravimetric and volumetric hydrogen content, borane‐based systems are being investigated as potential hydrogen‐storage materials for transportation technologies [370].

Beyond these applications, metallacarboranes have emerged as versatile functional materials with uses in nuclear waste remediation, advanced materials engineering, healthcare technologies, and energy‐related applications, largely owing to their remarkable stability and unique electron‐transfer characteristics [371].

## 5 Summary and Outlook

This review highlights how the human drive to understand and classify nature has shaped the development of chemistry, from early atomic theories to modern computational approaches. Within this broader context, it explores how aromaticity has evolved from a concept used to rationalize the stability and behavior of benzene and its derivatives into a general electronic‐structure framework applicable to diverse systems, including inorganic clusters, macrocycles, and excited states. Despite its widespread use, aromaticity remains inherently ambiguous, as different magnetic, electronic, geometric, and energetic descriptors often yield conflicting interpretations. The central message of this review is that the most informative cases often arise at the boundaries of classical definitions of aromaticity, where simple rules such as Hückel or Baird aromaticity become insufficient, and a more state‐, topology‐, and pathway‐aware interpretation is required.

Looking forward, the integration of computational chemistry, spectroscopy, and machine learning is expected to refine aromaticity descriptors and improve their predictive power. A reliable analysis should therefore combine complementary descriptors rather than rely on a single index. This is especially important for large, flexible, open‐shell, excited‐state, and 3D systems, where conformational effects, environmental interactions, vibronic contributions, and method dependence may strongly affect the interpretation. Advances in this direction will not only deepen fundamental understanding of electronic structure but also enable the rational design of functional materials, including optoelectronic devices, catalysts, and molecular medicines. Ultimately, aromaticity will continue to serve as both a conceptual tool and a practical guide in modern chemical research.

## Conflicts of Interest

The authors declare no conflicts of interest.

## Data Availability

Data sharing not applicable to this article as no datasets were generated or analyzed during the current study.
